# Bentonite-Clarified White Wine: Linking Clay Physico-Chemical Properties to Protein Removal Efficiency and Wine Matrix Alterations

**DOI:** 10.3390/molecules30204117

**Published:** 2025-10-17

**Authors:** Igor Lukić, Ivana Horvat, Doris Delač Salopek, Tajana Begović, Igor Djerdj, Stjepan Šarić, Vedrana Špada, Josipa Bilić, Igor Palčić, Zoran Užila, Smiljana Goreta Ban

**Affiliations:** 1 Institute of Agriculture and Tourism, Karla Huguesa 8, HR-52440 Poreč, Croatia; ihorvat@iptpo.hr (I.H.); doris@iptpo.hr (D.D.S.); palcic@iptpo.hr (I.P.); zoran@iptpo.hr (Z.U.); smilja@iptpo.hr (S.G.B.); 2 Department of Chemistry, Faculty of Science, University of Zagreb, Horvatovac 102a, HR-10000 Zagreb, Croatia; tajana@chem.pmf.hr; 3 Department of Chemistry, Josip Juraj Strossmayer University of Osijek, Cara Hadrijana 8/A, HR-31000 Osijek, Croatia; igor.djerdj@kemija.unios.hr (I.D.); stjepan.saric@kemija.unios.hr (S.Š.); 4 METRIS Research Centre, Istrian University of Applied Sciences, Preradovićeva 9D, HR-52100 Pula, Croatia; vspada@iv.hr (V.Š.); jbilic@iv.hr (J.B.)

**Keywords:** bentonite, winemaking, pathogenesis-related proteins, fining, cations, phenols, aroma compounds

## Abstract

Bentonites used for wine clarification vary widely in their ability to remove proteins and alter wine composition, yet the role of their intrinsic properties remains unclear. To address this, eight commercial bentonites with diverse physico-chemical characteristics were analyzed. The doses required for complete protein removal and stabilization were determined and then applied to clarify a Malvazija istarska (*Vitis vinifera* L.) white wine. Clarified wines were compared with one another and with a non-clarified control using ICP-OES for elemental composition, HPLC-DAD for phenolic compounds, and HS-SPME-GC/MS for volatile compounds. Protein removal efficiency correlated positively with Na/Ca ratio, cation exchange capacity, swelling capacity, negative *ζ*-potential, and internal specific surface area, and negatively with particle size and external specific surface area. Sodium and calcium showed the greatest increases in wine concentrations. Effects on individual low-molecular-weight phenols were inconsistent, though all bentonites removed a fraction of total phenols. Volatile compounds, particularly esters, were significantly reduced. When compared on a per-gram basis, bentonites that were more efficient in protein removal also showed greater removal of phenols and volatile compounds; however, at full application doses, many of these differences diminished or reversed. Overall, the study advances understanding of bentonite–wine interactions and supports more informed selection of bentonites in oenological practice.

## 1. Introduction

The clarification and protein stabilization of white wines through the removal of haze-forming proteins is an important step in winemaking aimed at ensuring visual stability and sensory quality during storage and consumption. Among available fining agents, bentonite remains by far the most widely used and effective material for this purpose, despite ongoing interest in alternative technologies [1,2,3,4,5,6,7,8,9].

Bentonite is a natural aluminosilicate clay, classified as a smectite of the montmorillonite type, featuring a laminar structure in which an octahedral alumina layer is “sandwiched” between two tetrahedral silica layers. Substitution of aluminum ions with other cations in these layers generates a net negative surface charge, which is neutralized by exchangeable cations, primarily Na^+^, Ca^2+^, and Mg^2+^, as well as other cations to a minor extent, located between the layers and on the outer surface. Upon application during clarification, these cations are exchangeable with those from wine, enabling electrostatic interactions with proteins which are positively charged at typical wine pH values (~3.0–3.6) and are subsequently removed from wine with sedimented bentonite [10,11,12,13]. According to the International Organisation of Vine and Wine (OIV), three types of bentonites are available for winemaking: natural sodium bentonite, natural calcium bentonite, and activated calcium bentonite, the latter modified by ion exchange with sodium carbonate [11,14]. Commercially, bentonite is sold in powder or granular form and must be hydrated in water before use. Hydration promotes swelling, which is critical for exposing the internal surface area and interlayer spaces. This swelling behaviour is strongly influenced by the composition of exchangeable cations, particularly the Na^+^/Ca^2+^ ratio, which affects the extent of interlayer expansion and, consequently, the clay’s adsorption capacity. The ratio of both major and minor cations can vary significantly between different bentonites [11]. The protein removal efficiency of bentonite is generally considered to correlate with its swelling capacity, surface charge density, and specific surface area, all of which depend on its mineralogical and physico-chemical characteristics [12,13]. Importantly, factors such as wine pH, temperature, and ethanol content also modulate bentonite’s performance by altering the charges and solubility of both wine proteins and bentonite particles themselves [15,16,17].

Despite its utility, bentonite’s adsorption behaviour is increasingly recognized as nonselective, often leading to the removal of desirable wine constituents alongside unstable proteins. An increasing number of studies have highlighted that bentonite treatment may inadvertently reduce the concentrations of phenolic compounds, volatile aroma compounds, and other molecules important for the physico-chemical and sensory quality and stability of wine [4,12,18,19,20,21,22,23]. For instance, research by Lambri et al. [18] demonstrated significant losses of odoriferous ethyl and acetate esters in white wines treated with bentonite after fermentation. Similarly, studies by He et al. [19] and Arenas et al. [24] reported the reduction in low-molecular-weight and total phenols, respectively, important for colour and oxidative stability of white wine. The cation exchange between bentonite and wine, as previously mentioned, was shown to significantly alter the wine’s elemental composition during treatment [11,17,23,25,26], potentially affecting its stability, health-related properties, and the reliability of geographical origin authentication.

Recent studies have highlighted significant variability among commercial bentonite products in protein removal efficiency and interactions with wine components [12,17,18,23,27,28]. Despite these observations, the role of bentonite’s intrinsic physico-chemical properties, such as cation composition and exchange capacity, swelling index, particle size, specific surface area, and surface charge in shaping its performance and associated side-effects remains poorly understood. This study systematically investigated the relationship between key physico-chemical properties of eight commercial bentonites and their effects on white wine, including protein removal efficiency and alterations in wine composition. By integrating bentonite characterization with detailed wine profiling, this research provides novel insights into the mechanisms underlying bentonite–wine interactions. The findings are expected to advance the scientific understanding of the bentonite clarification process and contribute to more informed selection of bentonites in oenological practice.

## 2. Results and Discussion

### 2.1. Bentonite Physico-Chemical Properties

One-way ANOVA revealed significant differences in the physico-chemical properties of the investigated bentonites (Table 1). The elemental composition, as determined by energy-dispersive X-ray spectroscopy (EDS), representing the near-surface region of the particles, revealed the dominance of silicon (Si), followed by aluminum (Al), the two main structural elements of bentonite tetrahedral and octahedral layers, respectively. A very strong negative correlation between Si and Al (Table S1) might have reflected tetrahedral Al^3+^/Si^4+^ substitution or the presence of accessory Si minerals such as quartz or opal-CT in particular samples [29]. Among the exchangeable cations, Na was most abundant in samples B2, B4, and B5 (Table 1). Its mass fraction was lower in B7, followed by B3 and B6, lower still in B1, and reached its minimum in B8. In contrast, Ca mass fraction peaked in B8, followed by B1 and B5, with the remaining samples showing lower and relatively uniform values. The Na/Ca ratio was the highest in B2 and B4, followed by B7, intermediate in B3, B5, and B6, and the lowest, mirroring the Na pattern, in B1 and especially B8. Although classified as a Na-Ca bentonite (Section 3.2), the Na/Ca ratio in B8 aligned more closely with that of a typical Ca-bentonite. The Na and Ca contents showed a strong negative correlation across the samples (Table S1). Bentonite B8 contained the highest Mg and Fe mass fractions (Table 1). The lowest Mg contents were found in bentonites B1, B2, and particularly B3, whereas Fe was least abundant in B1 and B4. The K mass fraction was the highest in B7 and the lowest in B4, B6, and B8. A significant negative correlation of Na with Mg, and a significant positive correlation of Ca with Mg (Table S1), might have reflected Na activation processes involving the replacement of exchangeable Ca^2+^ and Mg^2+^ by Na^+^. In addition to the original composition of exchangeable cations in smectite, the dominant mineral in bentonite, and subsequent natural or processing-induced alterations, the measured elemental composition may also reflect contributions from minor accessory minerals such as opal, quartz, plagioclase, calcite, halite, etc. For example, Catarino et al. [11] reported a positive correlation between Ca mass fraction and calcite content.

Based on the significant positive correlations (Table S1), the major-element composition (Si, Al, Na, Ca) of NH_4_Cl bentonite extracts agreed reasonably well with EDS results. However, the correspondence was not exact, indicating that the mineralogical structure and overall composition of the bentonites influenced the extractability and exchange behaviour of the cations. Similar outcomes were noted by other authors [11]. Although Si and Al are structural components of bentonite layers and generally non-exchangeable, the data indicated that some leaching occurred during extraction. The highest Si concentration was found in the extract of B3 (Table 1), consistent with its declaration of containing adsorbed silica gel and activated silica (Section 3.2). Na concentration was the highest in B5, followed by B6. B1 contained less Na than all the other bentonite extracts except B8, which had by far the lowest Na concentration. In contrast, Ca was most abundant in B8, followed by B1 and B4. Consequently, the Na/Ca ratio was the highest in B5 and B6, and the lowest in B1 and especially B8. Concentrations of other cations in the NH_4_Cl extracts did not correspond closely to their mass fractions determined by ESD. Magnesium concentration was the highest in B6 and the lowest in B1. Potassium was most abundant in B2, followed by B4 and B1, and the lowest in B3 and B8. Relatively high Mn concentrations were observed in B1 and B3 extracts.

Bentonites B5 and B6 had the highest cation exchange capacity (CEC), followed by B7 (Table 1). The lowest CEC values were determined for B1 and especially B8. CEC showed a strong positive correlation with Na concentration and the Na/Ca concentration ratio, and a slightly weaker but still positive correlation with Na mass fraction and the corresponding Na/Ca ratio. A moderate negative correlation was observed between CEC and Ca, both in mass fraction and concentration (Table S1). The highest swelling capacity was observed in B5, followed by B2 and B4 (Table 1). Bentonites B6 and B7 showed lower swelling, B1 even less, and the lowest was recorded for B8. The swelling capacity of the bentonites showed a strong positive correlation with their Na content, the Na/Ca ratio (both mass ratio and extract concentration), and CEC (Table S1). These results align with the widely recognized fact that Na-rich bentonites exhibit greater interlayer expansion due to the high hydration potential of Na^+^, which promotes swelling and increases the accessibility of surface charge sites for ion exchange. In contrast, Ca-dominated bentonites are less swellable because Ca^2+^ binds more strongly between layers, restricting expansion and thereby limiting their CEC [30,31,32]. However, evidence shows that increasing Na (or Na/Ca) does not necessarily increase CEC and may even result in plateaus or declines at high Na activation [33].

Particle size distribution analysis by static light scattering (SLS) showed that B8 had the largest particle diameter, followed by B1 and B3 (Table 1). B6 had smaller particles, B4 and B7 even finer, and the smallest were observed in B2 and B5. Particle size correlated positively with Ca content and negatively with Na content, the Na/Ca ratio, CEC, and swelling capacity, consistent with reports that Na-dominated montmorillonites tend to delaminate extensively in suspension and form smaller, more mobile colloids, whereas Ca-dominated types form larger aggregates [34,35,36].

The bentonites were clearly differentiated by external specific surface area (E-SSA), determined by N_2_ adsorption using the BET method (Table 1). B8 exhibited the highest E-SSA. It was followed by B1, while B2 and B3 were characterized by slightly lower but similar values. Among the remaining samples, E-SSA decreased in the order B6 > B7 > B4 > B5. E-SSA correlated positively with particle size and negatively with Na content, Na/Ca ratio, CEC, and swelling capacity (Table S1). This was consistent with earlier studies reporting that Na-bentonites exhibit lower E-SSA than Ca-bentonites [35,37]. Catarino et al. [11] observed that the lowest E-SSA coincided with the highest CEC across various bentonites. Kaufhold et al. [35] argued that E-SSA is controlled chiefly by crystallochemical parameters, namely composition and layer-charge density. They further proposed that Ca-bentonites exhibit higher E-SSA than Na-bentonites because Ca^2+^-saturated interlayers retain more free volume and microporosity. To balance the permanent negative layer charge, only half as many Ca^2+^ ions as Na^+^ ions are needed, so fewer interlayer sites are occupied, and more N_2_-accessible interlayer space (especially at edges) remains. In contrast, Na-bentonites require twice as many Na^+^ ions, which crowd interlayer regions and partially block N_2_ access, lowering the measured E-SSA. The authors further noted that variations in the number of collapsed layers among bentonites can occlude edge interlayers, rendering these sites inaccessible to N_2_ [35].

Unlike N_2_-BET analysis, which measures the external surface area and only a fraction of microporosity, the methylene blue (MB) adsorption test, performed in aqueous suspension, primarily quantifies the internal specific surface area (I-SSA) of bentonite [38,39]. Some authors interpret MB uptake as a measure of total (external + internal) surface area [40]. Swelling in water renders interlayer sites accessible, so in Na-rich bentonites the more expanded layers allow MB to reach interlayer binding sites more readily than in the relatively rigid or narrow interlayers typical of Ca-forms [30,41,42]. In this study, the highest I-SSA was found for B2 and B5, followed by B7, with values further decreasing in the order B4 > B8 > B6 > B3 > B1 (Table 1). Significant positive correlations were found between I-SSA and Na content, the Na/Ca ratio, CEC, and swelling capacity (Table S1), consistent with prior reports linking MB-derived surface area to CEC and swelling potential across mineral soils [40,43]. However, the observed correlations were mostly moderate, consistent with previous studies on heteroionic bentonites used in wine clarification, which reported inconsistent relationships between I-SSA, E-SSA, Na content, and CEC [11,12,17,44]. Such findings suggest that additional mineralogical factors and solution conditions can significantly influence the outcomes.

Bentonites were grouped into two clusters based on their *ζ*-potential, reflecting the differences in their surface charge (Table 1). Samples B4, B5, and B6 exhibited lower values than the others, indicating more negatively charged layer faces. The exception was B7, which showed an intermediate value. The measured *ζ*-potential was clearly related to Na content, Na/Ca ratio, CEC, and swelling capacity, as significant correlations were observed between these properties (Table S1). This supported the hypothesis that the dominance of monovalent Na^+^ in the interlayer space enhanced dispersion and exposed the permanent negative charges arising from isomorphic substitution in the montmorillonite layers, thereby lowering the *ζ*-potential. This was also in line with previous findings showing that *ζ*-potentials of relatively pure Na-bentonites are generally more negative than those of Ca-bentonites [45,46]. However, the correlation found in this study was only moderate because *ζ*-potential reflects not just Na content or CEC, but also pH-dependent edge charges, ionic strength, cation valence, charge location, octahedral chemistry, etc., which together obscure a direct proportionality [45,46].

**Figure 1:**
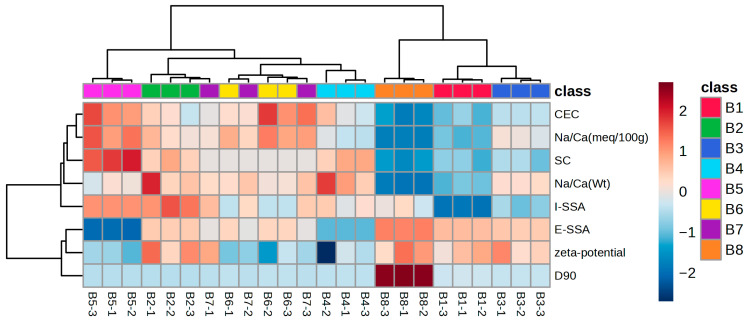
Hierarchical clustering analysis of different bentonites (B1–B8) based on their physico-chemical properties. The rows in the heatmap diagram represent physico-chemical properties, and the columns represent bentonite samples. Sample codes combine the bentonite type and replicate number (e.g., B1-1 for B1 in the first replicate, B1-2 for the second, etc.). Abbreviations: CEC—cation exchange capacity; SC—swelling capacity; I-SSA—internal specific surface area; E-SSA—external specific surface area; D90—diameter at which 90% of the particle volume is smaller. Cell colouring encodes normalized abundance: low (dark blue), medium (pale tones), and high (dark red).

Hierarchical clustering analysis (HCA) was performed to visualize differences and similarities among the bentonites based on their physico-chemical properties (Figure 1). Bentonites B1, B3, and B9 formed a distinct cluster, characterized by lower Na/Ca ratio, CEC, swelling capacity, and I-SSA and higher E-SSA, particle size, and (i.e., less negative) *ζ*-potential. B5 and B4 were farthest from the B1–B3–B9 cluster and showed the inverse pattern: higher Na/Ca ratio, CEC, swelling capacity, and I-SSA, more negative *ζ*-potential, and lower E-SSA and particle size. Although these variables generally co-varied, they did not do so perfectly; thus, the separation reflected their combined multivariate structure rather than any single variable. The remaining bentonites (B2, B6, B7) joined B5 and B4 at a lower linkage height, indicating greater similarity to that group than to B1, B3, and B9. Samples B2, B6, and B7 did not resolve into discrete sub-clusters by type, indicating broadly similar property profiles.

### 2.2. Protein Removal Efficiency and Bentonite Lees Sedimentation

Bentonite’s negatively charged montmorillonite layers provide exchange sites that adsorb positively charged haze-forming proteins at wine pH and form clay–protein complexes that flocculate and settle as lees. These processes involve cation exchange, electrostatic forces, hydrogen bonding, and hydrophobic dispersive interactions, occurring on the interlayer surfaces, the edges of the clay, or both [47,48]. According to Schoonheydt and Johnston [48], proteins adsorb rapidly at particle edges and intercalate where interlayers are accessible. These authors showed that Na-saponite adsorbs markedly more protamine than low-swelling Cs-saponite and presumed that intercalation becomes harder with increasing protein molecular weight and lower net positive charge. Consistently, it was shown that lysozyme binds mainly to Cs-saponite external surfaces because interlayer access is limited [49]. A model-wine study showed that ethanol increases interlayer accessibility and enhances the adsorption of smaller proteins [50]. Significant intrusion of protein chains into clay interlayers may even cause exfoliation or delamination of the silicate layers [47]. Overall, it is evident that the balance between edge and interlayer binding and protein removal efficiency depend on exchangeable-cation composition (Na^+^/Ca^2+^ ratio), hydration/swelling capacity, and other bentonite properties, as well as ethanol content, and protein size and charge. This suggests that, during wine clarification, protein adsorption occurs on bentonite edges with variable interlayer participation, depending on bentonite type and other conditions.

The tested bentonites required markedly different doses to achieve complete protein removal and stabilize Malvazija istarska white wine (Table 1). A subset of bentonites showed relatively similar requirements, ranging from 160 to 220 g/hL: B4 required the lowest dose, followed by B6, B2, B5, and B7 in increasing order. The remaining three required substantially more: B3 at 300 g/hL, B1 at 380 g/hL, and B8, which required the highest dose by far at 650 g/hL. These groupings mirrored the HCA results (Figure 1) and were consistent with a causal link between bentonite properties and protein-removal efficiency, that is, the dose required for complete protein stabilization. Significant, very strong negative correlations were observed between bentonite dose and Na content, the Na/Ca ratio, CEC, and swelling capacity, along with a moderate negative correlation with the magnitude of the negative *ζ*-potential (Table S1). These results clearly indicate that bentonites with higher values of these parameters exhibited greater protein removal efficiency. In contrast, dose showed strong positive correlations with Ca content, particle size, and E-SSA. Inverse relationships were found for bentonite sediment, marked by a strong negative correlation with dose. The proportions of sediment in clarified wine correlated moderately to strongly and positively with Na content, the Na/Ca ratio, CEC, swelling capacity, and the magnitude of the negative *ζ*-potential, and negatively with Ca content, particle size, and E-SSA. Only a moderate positive correlation determined between swelling capacity and sediment volume indicates differences in settling behaviour among the bentonites, particularly influenced by the wine matrix. The observed lower required doses and greater sediment volumes for Na-richer (e.g., B5 and B6) compared to Ca-richer bentonites (e.g., B1 and B8) were consistent with earlier findings and with practical winemaking experience [15], although the relationship was not completely linear.

Based on the results obtained, most bentonite properties, particularly its efficiency in protein removal and sediment volume, appeared to be tightly related to the quantities and ratio of its main exchangeable cations, Na^+^ and Ca^2+^. Due to their lower ionic potential (charge-to-size ratio), Na^+^ ions bind less strongly in the interlayers and have thinner hydration shells than Ca^2+^. As a result, Na-rich bentonites display greater swelling, cation exchange capacity, and dispersibility with delamination, exposing more surface area and exchange sites than Ca-bentonites. This increases protein adsorption at wine pH, allowing stability to be achieved with lower clay doses. In contrast, Ca-bentonites swell less, limiting access to interlayer sites. They tend to form denser, larger aggregates that settle efficiently but remove proteins less effectively per unit mass, necessitating higher additions [12,30,31]. Because bentonites used in wine clarification are often heteroionic and wine generally has an ionic strength distinct from clay–water suspensions, the relationship between their physico-chemical properties and protein removal efficiency is more complex. Protein removal efficiency depends strongly on overall wine composition, particularly pH and ethanol content, which influence protein charge as well as bentonite deproteinization, surface charge, and swelling behaviour [17,18,50]. Furthermore, because bentonite activity is not selective, other wine components, such as phenols, polysaccharides, and metal cations, also compete with proteins for binding to its active sites, which may affect the final clarification efficiency [11,13,47]. Several studies have reported no strictly linear relationship between Na^+^ and Ca^2+^ contents, related bentonite properties, and protein removal efficiency [17,44,51], underscoring the complexity of these interactions. For example, Lambri et al. [18] found no clear correlations between protein removal efficiency, Na/Ca, and I-SSA, and inferred that structural properties (e.g., SSA) are more decisive than the Na/Ca ratio for protein-removal performance of a given bentonite. In a subsequent study on a different set of bentonites, they observed that the highest Na/Ca ratio coincided with the highest I-SSA [12]. It is worth noting that the present study included bentonites with markedly different properties, particularly B1 and, most notably, B8, which likely contributed to the observed differences and intercorrelations.

### 2.3. Alterations in Elemental Composition

In wine clarification, the mechanism responsible for protein removal is considered to be based, for the most part, on cation exchange. Exchangeable cations in bentonite, primarily Na^+^ and Ca^2+^, are displaced by positively charged protein molecules, enabling their adsorption onto the clay and release of cations into wine [11]. This same mechanism also drives broader changes in wine’s elemental composition. Bentonite-derived cations can exchange with abundant wine cations such as K^+^, while leaching of structural minerals (Si, Al, Mg) can additionally raise their concentrations in the finished wine. At the same time, bentonite can reduce the levels of certain elements through more complex mechanisms. For example, the depletion of B, Cu, and Zn has been attributed to their complexation with tannins and polysaccharides, which are subsequently removed together with the clay [11]. The extent of these exchanges and transfers is shaped by bentonite properties and wine pH, ionic strength, and composition [11,17,23,26,51].

Bentonite clarification treatments significantly altered the wine matrix with respect to elemental composition (Table 2). In most cases where changes were statistically significant, element concentrations increased. For macroelements such as K, P, and S, changes were generally mild. Potassium content increased by 4.53% in B2 compared to C and was also higher in B2 than in B8. Magnesium levels increased in B7 and B8 by 10.2% and 11.1%, respectively, relative to C. B8 wine had a significantly higher Mg concentration than all treatments except B5 and B7, while B7 contained more than B1–B4 wines. A moderate, statistically significant positive correlation was observed between the change in wine Mg concentration and the Mg mass fraction of the bentonites (Table S2). Calcium showed more pronounced changes. Relative to the control C, treatment B8 increased wine Ca concentration by 83.5% and B1 by 41.3%. These effects were consistent with the highest Ca mass fractions in these bentonites and the highest Ca concentrations in their NH_4_Cl extracts (Table 1), which was supported by significant positive correlations between wine Ca and bentonite (extract) Ca content (Table S2). Wine Ca concentration correlated negatively with Na and with the Na/Ca ratio in both bentonite mass fractions and extract concentrations. All bentonite treatments markedly increased Na content, ranging from a 165% rise in B8 to 301% in B3 (Table 2). Notably high Na levels were also observed in B5 and B7, while B6 showed a relatively lower increase, similar to B8. On a dose-normalized basis (per gram of bentonite applied), the increase in wine Na concentration correlated positively with both the Na mass fraction of the bentonites and the Na concentration in NH_4_Cl extracts (Table S2). In contrast to the changes observed for wine Ca, this relationship was not evident without dose normalization. This suggests that, for bentonite-induced alterations in wine elemental composition, dose may exert a more decisive effect than the bentonite’s cation composition or cation-exchange capacity.

Treatments B1–B3 and, most notably, B7 led to increased Al content (Table 2). Iron concentration was higher in B5, B7, and B8 compared to B6. Most bentonite treatments tended to increase Fe concentration relative to C but did not reach statistical significance. Wine Mn concentration increased with treatments B7 and B4, and most strongly with B1 and B8. It correlated positively with Mn in NH_4_Cl extracts, driven by the high extractable Mn from B1, B4, and B8. Wine Mn also correlated strongly with Ca (positive) and Na (negative) in both bentonite mass fractions and NH_4_Cl-extract concentrations, suggesting that Mn may be hosted in Ca-bearing carbonates (e.g., calcite) in such Ca-richer bentonites [11,52]. A significant decrease in Zn levels was observed in wines treated with B3 and B6, and the majority of other treatments showed a similar tendency. Cobalt concentrations in the treated wines did not differ significantly from that in C, with B2 and B8 having higher levels than B5 wine.

The observed alterations in elemental composition across most treatments were consistent with previously reported increases in Ca, Na, Al, and Mn and decreases in Zn concentration in wine following bentonite application [11,23,25,26,53]. While most authors also reported increases in Mg, Fe, and Co and decreases in K [11,25,26,53], these trends were observed only occasionally or not at all in this study. Exceptions have also been noted in previous works, for example, no change in Ca concentration when Na-activated Ca bentonites were used, attributed to the replacement of Ca with Na ions during activation [53], variable Fe responses depending on bentonite dose [26], increases in K [23] and Zn [53], etc. Particularly noteworthy are the findings of Dordoni et al. [17], who reported that the direction and magnitude of Na and Ca concentration changes varied significantly with wine pH and the specific bentonite used. Changes in the wine’s elemental composition only partially mirrored the composition of the bentonites and/or their NH_4_Cl extracts: relationships were strongest for the major cations Na and Ca, followed by Mg, Fe, and Mn, whereas no direct links were detected for other elements (Table 2 and Table S2). A similar lack of complete correspondence was reported by other authors [11,51]. Despite recurring trends, the effects of bentonite treatment on wine elemental composition obviously depend strongly on bentonite type and properties, dosage, application method, and initial wine composition.

### 2.4. Alterations in Phenolic Compound Composition

Phenolic compounds in wine are mostly neutral but polar due to uneven electron distribution, enabling interactions with bentonite through mechanisms beyond electrostatic binding, such as Van der Waals forces, hydrogen bonding, coordination, and chemisorption [12,15,50]. Bentonite clays have a much lower affinity for phenolics than for proteins [17,54], as confirmed recently by adsorption free energy measurements [21]. However, protein deposition on clay surfaces can alter surface properties, promoting multilayer adsorption and enhancing phenol binding [17,55]. Jaber et al. [56] hypothesized that ion exchange, in which proteins replace bentonite cations within the interlayer spaces, alters the surface chemistry of the interlayers, converting their initially hydrophilic character into a more hydrophobic one. This transformation could reduce electrostatic repulsion between bentonite and phenols, create sites for hydrophobic dispersive interactions, and enable secondary adsorption of phenols onto proteins already bound to the clay. In a study using model wine, Trigueiro et al. [47] demonstrated that bentonite can bind phenols (such as resveratrol) either directly, primarily on the clay’s surface and edges, or indirectly through complexation with proteins. Pargoletti et al. [21] observed that flavan-3-ol monomers and dimers were removed by bentonite over three times more in the presence of egg albumin, likely due to hydrogen bonding between phenols and proteins. Thus, the impact of bentonite clarification on phenols likely depends on interactions between bentonite and wine proteins.

Clarification with different bentonites significantly altered the composition of phenolic compounds in the investigated wine (Table 3). The effects varied depending on the specific bentonite and compound involved. Treatment with B1 increased the levels of protocatechuic and *p*-hydroxybenzoic acid, while treatments B6 and B8 reduced the levels of 2,5-dihidroxybenzoic acid. Although differences were noted among the various bentonite treatments, the changes in hydroxybenzoic acid concentrations were generally limited, not exceeding 10% in most cases. He et al. [19] found that bentonite clarification reduced gallic acid in both Chardonnay and Sauvignon Blanc, while vanillic acid decreased only in Sauvignon Blanc. Other hydroxybenzoic acids remained unchanged.

Among hydroxycinnamoyltartrates, the concentration of the most abundant compound, *trans*-caftaric acid, was significantly increased by all treatments except B6 and B7. Several treatments also increased the level of *cis*-coutaric acid. A similar trend was observed for the less abundant isomers, with *trans*-coutaric acid showing a significant increase across all bentonite treatments, ranging from 13.7% in B5 to 21.6% in B8. Likewise, several clarification treatments showed a tendency to increase the concentrations of both fertaric acid isomers. Although limited in number, earlier studies have reported somewhat inconsistent results regarding the interactions between bentonite and hydroxycinnamoyltartrates. He et al. [19] reported a decrease in *trans*-caftaric acid after clarification with various bentonites, while Arenas et al. [24] found no changes in caftaric or coutaric acid levels after clarification with either sodium or calcium bentonite. Interestingly, the same authors reported no effects, increases, and decreases in total non-flavonoid phenols, depending on the wine style and type of bentonite used. Hydroxycinnamoyltartrates are esters formed from hydroxycinnamic acids and tartaric acid. According to the Human Metabolome Database [57], caftaric acid’s most acidic carboxyl group has a pKa of 2.96. At the wine’s pH, it is therefore partially deprotonated and exists in its anionic form (>50%), while the remainder remains neutral, suggesting it is less prone to direct electrostatic binding to the negatively charged bentonite.

The higher concentrations of certain hydroxycinnamoyltartrates in bentonite-treated wines, compared to the control, may be due to several factors. One possibility is that residual hydroxycinnamoyl esterase activity was greater in the control wine C, since the enzyme may have been adsorbed by bentonite in the treated samples. Another explanation could be the release of these compounds from complexes following changes in ionic strength caused by bentonite. Alternatively, bentonite might have removed components that normally bind to hydroxycinnamoyltartrates. However, these are only hypotheses, as no supporting evidence is currently available in the scientific literature. On a dose-normalized basis, certain hydroxycinnamoyltartrates showed positive correlations with bentonite Na content, the Na/Ca ratio, CEC, swelling capacity, and applied dose, and negative correlations with Ca content, particle size, E-SSA, and sediment formation (Table S3). These patterns suggest that bentonite properties exert a more systematic influence, with Na-richer bentonites tending to better preserve these phenolic compounds during clarification on a per-gram basis.

Changes in free hydroxycinnamic acids were less pronounced. The concentrations were generally comparable to those in the untreated control wine C, and statistically significant differences were observed in only a few cases (Table 3). Unlike hydroxycinnamoyltartrates, free hydroxycinnamic acids such as caffeic, *p*-coumaric, and ferulic are mostly neutral at wine pH, as the pKa of their carboxylic group is around 4.4 [58]. Consequently, differences in their interaction with bentonite can be expected. Caffeic and *p*-coumaric acid levels correlated positively with swelling capacity and I-SSA and negatively with E-SSA (Table S3). A previous study by Arenas et al. [24] found that clarification with either sodium or calcium bentonite did not significantly alter free hydroxycinnamic acid levels. In contrast, He et al. [19] reported that most of the four bentonites tested reduced caffeic and *p*-coumaric acids, while ferulic acid remained unaffected.

Epicatechin levels were higher in B1 than in B8, though none of the treatments caused a significant change relative to control C wine. B1 caused a 25.8% reduction in procyanidin B1, while B4–B8 showed a tendency to increase its concentration. Procyanidin B2 levels were elevated in treatments B4–B7 compared to the control C. Per gram of bentonite, procyanidins B1 and B2 were positively associated with Na content, the Na/Ca ratio, CEC, swelling capacity, negative *ζ*-potential, I-SSA, and sediment volume, and negatively with E-SSA and dose (Table S3). These relationships suggested a potential influence of bentonite composition and properties, although the effect was obscured when different doses were applied. In a previous study, clarification led to variable effects on specific flavan-3-ol monomers, dimers, and their hydroxylated and galloylated derivatives, depending on the wine variety and bentonite type used [19]. A similar trend was observed by Arenas et al. [24] for total flavonoid phenols. Among stilbenes, no significant differences were observed for *cis*-piceid, while all treatments except B4 led to an increase in the concentration of the *trans* isomer (Table 3).

All bentonite treatments reduced total phenol content, ranging from an 8.1% decrease with B4 to a maximum reduction of 13.0% with B5 (Table 3). The reduction in absolute concentrations was much greater than that observed for individual low-molecular-weight phenolic compounds identified by HPLC. This likely reflected the removal of higher-molecular-weight species, such as flavan-3-ol oligomers and polymers (i.e., condensed tannins or proanthocyanidins), which were not specifically analyzed in this study. Although tannins are much less abundant in white wines than in reds, they still represent a significant portion of the phenolic content with concentrations ranging from 20 to 80 mg/L [59]. Tannins are expected to interact more readily with bentonite than low-molecular-weight phenolics due to their multiple aromatic rings and polarizable groups, offering greater surface area and more potential binding sites for the charged clay layers and hydrophobic regions. On a per-gram basis, bentonites with higher Na content, Na/Ca ratio, CEC, swelling capacity, and more negative *ζ*-potential showed greater removal of total phenols. In contrast, the removal was inversely related to Ca content, particle size, E-SSA, and sediment volume (Table S3). It is probable that, as with their interaction with proteins, Na-richer bentonites, characterized by greater cation exchange capacity, swelling, and dispersibility with delamination, exposed more surface area and exchange sites for phenol binding than Ca-rich bentonites. It is also likely that higher protein adsorption per gram on Na-richer bentonites provided additional surface for second-layer phenol binding through non-electrostatic interactions. Arenas et al. [24] reported either no change or a reduction in total phenols after bentonite clarification, depending on the wine style, with significantly greater reductions observed with Na-bentonite (18.6%) compared to Ca-bentonite (11.9%). Similarly, other studies have mostly reported decreases in total phenol concentrations, ranging from 1.3% to 21.9%, depending on the bentonite type, dosage, and the specific wine treated [17,19,23,24,60].

### 2.5. Alterations in Volatile Compound Composition

Similar to phenols, volatile compounds can be removed from wine during clarification either by direct adsorption onto bentonite or indirectly through binding to proteins that are subsequently removed. These interactions, both physical and chemical, depend on the bentonite type, dose, and wine matrix, and vary by compound based on molecular structure, hydrophobicity, and affinity for proteins or bentonite surfaces [4,12,18].

Bentonite clarification treatments resulted in significant changes to the wine’s volatile composition, with most statistically significant shifts corresponding to decreased concentrations (Table 4). Among terpenoids, linalool, the most sensorially relevant compound from this group, was unaffected by the clarification treatments. Minor changes were observed for geraniol, whose level was higher in B3 than in B7, and for 6-methyl-5-hepten-2-ol, which was higher in B3 than in B5 and the control C wine. The concentration of the only identified C_13_-norisoprenoid, β-damascenone, was significantly decreased by all treatments, with reductions ranging from 10.7% in B6 to 18.1% in B8.

C_6_-alcohols showed minor changes: 1-hexanol and *trans*-3-hexen-1-ol remained unaffected, while *cis*-3-hexen-1-ol showed a general increase, although the change was statistically significant only with B3. 2-Phenylethanol concentration was significantly reduced by B2, B7, and B8, and was higher in B3 than in B2, B4, and B6–B8. Hexanoic acid levels were significantly reduced by five of the eight treatments, with reductions ranging from 21.1% in B4 to 25.3% in B6. In contrast, most bentonites did not affect octanoic acid, except for a significant increase with B3.

Ethyl propanoate was the only ethyl ester unaffected by bentonite treatments. Clarification generally reduced the levels of other short-chain esters, such as ethyl isobutyrate and ethyl butyrate. Both esters had the lowest levels in B1 wine, though significant effects were sporadic. The concentration of ethyl 2-methylbutyrate was significantly decreased by all treatments except B6. Bentonites B2 and B5 decreased the level of ethyl 2-methylbutyrate. All treatments tended to reduce the concentrations of major medium-chain ethyl esters. Ethyl hexanoate decreased significantly with all treatments except B4 and B6, ranging from 13.0% in B2 to 21.1% in B1. Ethyl octanoate was significantly reduced by B1, B3, and B8. Ethyl decanoate levels showed the most pronounced decline, with all bentonites causing reductions from 14.5% in B6 to 48.3% in B7. Decreases in ethyl nonanoate were also consistent across all treatments, ranging from 16.2% in B8 to 29.1% in B2.

Among acetate esters, methyl and propyl acetate underwent sporadic alterations, mostly reductions. Isobutyl acetate concentrations were significantly lowered by B1–B3 and B8, while butyl acetate was reduced by all bentonite treatments, ranging from a 28.9% decrease in B7 to 42.0% in B6. Although all bentonites tended to reduce isoamyl acetate, a major ester in this group, only B1 and B4 caused significant reductions. A similar pattern was observed for C_6_-alcohol acetates: hexyl acetate was significantly reduced by B1, B2, and B7, *cis*-3-hexen-1-yl by B1, B2, B4, B5, and B7, and *trans*-3-hexen-1-yl only by B2. Bentonites B4, B6, and B7 significantly reduced 2-phenethyl acetate concentration.

Like its ethyl analogue, methyl decanoate showed significant depletion after all bentonite treatments, ranging from 21.8% with B2 to 48.2% with B8. Diethyl succinate responded variably, with a significant reduction for B2 and a notably large decrease of 38.2% with B8.

Correlation analysis revealed that methyl decanoate and diethyl succinate were more strongly removed at full doses with Ca-richer bentonites, and propyl acetate with Na-richer bentonites (Table S4). On a per-gram basis, however, bentonites with higher Na/Ca ratio, swelling capacity, CEC, and I-SSA, smaller particle size, and lower E-SSA generally showed greater affinity for adsorption and removal of volatile compounds, including β-damascenone, 2-phenylethanol, hexanoic acid, and several esters (Table S5). These results align with Lambri et al. [12], who found that bentonites with higher Na/Ca ratios and I-SSA showed greater affinity for volatile compound adsorption and removal, especially esters. Overall, the findings indicate that, similar to bentonite–protein interactions, the higher surface area and greater number of exchange sites in Na-richer bentonites promote stronger adsorption of most volatile compounds. Nonetheless, these relative differences appeared to be greatly reduced, annulled, or even reversed when full bentonite doses were applied.

Previous studies report widely varying effects of bentonite clarification on wine volatile compounds. Regarding varietal aroma compounds, bentonite had little to no impact on monoterpenes [4,61], consistent with the present findings, though some authors reported significant reductions in linalool [20]. While Vela et al. [61] and Ma et al. [20] found no effect on β-damascenone, Vincenzi et al. [4] observed a significant decrease in the related C_13_-norisoprenoid, 3-hydroxy-β-damascone. Findings for C_6_-alcohols were also inconsistent, ranging from negligible effects [4,18,20,61] to substantial losses [18]. Volatiles produced during fermentation showed the most variability and the greatest losses. Lambri et al. [18] reported changes from negligible to strong reductions in key fermentation aroma alcohols, fatty acids, ethyl and acetate esters, depending on bentonite type, dosage, wine matrix, and compound. Other studies also reported significant and variable losses [4,12,20,23,62]. In contrast, Vela et al. [61] found no significant decreases in major volatile alcohols, acids, and esters after clarification with bentonite at 100 g/hL.

Vincenzi et al. [4] found that the removal of fatty acids and their ethyl esters by bentonite during clarification of a model wine solution increased with the length of their hydrophobic straight alkyl chain. They attributed this to electrostatic repulsion between the negatively charged bentonite and the more polar, shorter-chain compounds, as well as enhanced affinity for the more hydrophobic, longer-chain molecules. Although medium-chain fatty acids are mostly protonated and thus neutral at wine pH due to their pKa values being above 4.8 [63], their polar carboxylic group can still engage in dipole interactions or hydrogen bonding or be repelled by highly charged regions depending on orientation. Longer-chain acids and esters, being more hydrophobic, likely interact more strongly with bentonite via van der Waals and dispersion forces, particularly in hydrophobic microdomains. In aqueous solutions, the lower solubility of hydrophobic compounds causes them to be ‘driven out’ of the bulk water phase toward surfaces, which may further enhance their affinity for adsorption onto bentonite [64]. Lambri et al. [12] reported a comparable relationship between hydrophobicity and adsorption for ethyl esters on certain bentonites in a model solution. However, the same compounds, particularly ethyl octanoate, displayed the opposite trend with a different type of bentonite. The authors concluded that adsorption was influenced more by the characteristics of the clay than by the properties of the compounds. Vincenzi et al. [4] demonstrated that the adsorption and removal of fatty acids and ethyl esters, particularly long-chain compounds, were enhanced in the presence of proteins, supporting the hypothesis that these aroma compounds bind to proteins, likely at hydrophobic regions, which are then removed by bentonite. In this study, the removal rate of straight-chain ethyl esters generally increased with alkyl chain length across all bentonites, except for ethyl octanoate. In contrast, hexanoic acid showed a significantly higher stripping rate than octanoic acid (Table 4). Other studies reported inconsistent relationships between fatty acid and ethyl ester alkyl chain lengths, and thus hydrophobicity, and their adsorption behaviour [18,20,23]. A recent study reported that certain transition metal cations, such as Fe, Mn, and Cu, can catalyze the hydrolysis of volatile esters in wine under specific conditions [65]. Although direct correlations were not established in this study, the possibility that cations leached from the investigated bentonite samples contributed to the hydrolysis of esters should not be completely overlooked.

### 2.6. Hierarchical Clustering Analysis of Wine Matrix Alterations

**Figure 2:**
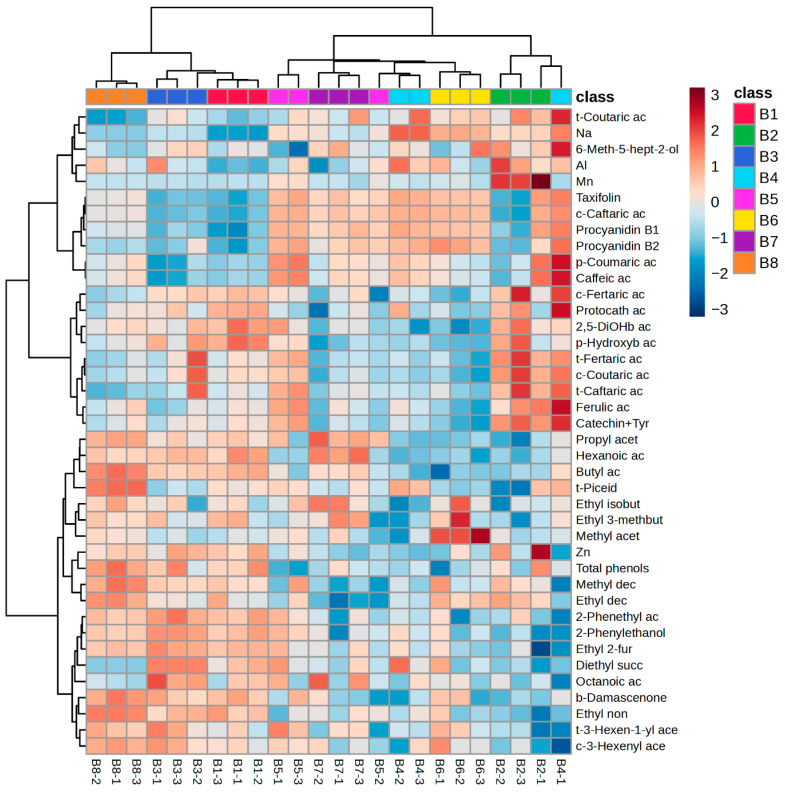
Hierarchical clustering of Malvazija istarska wines clarified with different bentonites (B1–B8), using relative concentration changes in elements, phenolic compounds, and volatile compounds normalized per gram of bentonite (vs. the control). The rows in the heatmap diagram represent physico-chemical properties, and the columns rep-resent bentonite samples. Sample codes combine the bentonite type and replicate number (e.g., B1-1 for B1 in the first replicate, B1-2 for the second, etc.). Cell colouring encodes normalized abundance: low (dark blue), medium (pale tones), and high (dark red).

To set aside the confounding effect of bentonite dose and more clearly reveal the intrinsic impact of its physico-chemical properties, HCA was performed using per-gram, treatment-normalized alterations of wine constituents as variables. HCA basically corroborated the links between bentonite properties and the effects of clarification on elemental, phenolic, and volatile composition of wine established by ANOVA (Figure 2). Wines clarified with B1, B3, and B8, characterized by lower Na/Ca ratio, CEC, I-SSA, and swelling capacity, a less negative *ζ*-potential, larger particle size, higher E-SSA, and weaker protein-removal efficiency (Table 1; Figure 1), clustered together and showed lower concentrations of Na, Al, and Mn, as well as several low-molecular-weight phenolic acids and procyanidins. The remaining bentonites formed two sub-clusters differentiated by their effects on other phenolics, with some wines exhibiting higher and others lower concentrations relative to the B1–B3–B8 group. Despite this internal split, all non-B1-B3-B8 bentonites grouped together and were separated from that cluster by lower *trans*-piceid and Zn levels and by greater stripping of multiple volatile compounds.

## 3. Materials and Methods

### 3.1. Wine Sample

The wine sample used for the study was produced from grapes of Malvazija istarska (*Vitis vinifera* L.), the most widespread and economically important native white cultivar in Croatia, harvested in 2021 from the experimental vineyard of the Institute of Agriculture and Tourism in Poreč (Istria, Croatia). The physico-chemical parameters of the wine, determined by OIV methods, were as follows: alcoholic strength 13.1 vol.%, total dry extract without reducing sugars 19.8 g/L, total acidity 5.9 g/L (as tartaric acid), volatile acidity 0.32 g/L (as acetic acid), and pH 3.24. The concentration of total proteins, determined according to the protocols established by Marangon et al. [66] and Van Sluyter et al. [67], and described in Lukić and Horvat [68], was 225 ± 3 mg/L (*n* = 3).

### 3.2. Bentonite Samples

Eight bentonites from different producers available from various suppliers in Croatia were characterized and tested in clarification trials. Specifications of type and form provided by producers were as follows: B1: Na-activated, powder; B2: Na-activated, granular; B3: Na-activated with silica gel/activated silica, granular; B4: Na-activated, pellets; B5: Na-activated, powder; B6: Na-activated, granular; B7: Na natural, granular; B8: Na-Ca, granular.

### 3.3. Physico-Chemical Analysis of Bentonites

#### 3.3.1. Elemental Composition

The elemental composition of the bentonite samples was determined by energy-dispersive X-ray spectroscopy (EDS) with a scanning electron microscope (SEM). Prior to analysis, samples were dried to constant mass, gently ground, and fixed onto conductive carbon tape. Measurements were conducted using a QUANTA 250 FEG microscope (FEI Company, Hillsboro, OR, USA) fitted with an Oxford PentaFET EDS detector (Oxford Instruments, Abingdon, UK), operating at an accelerating voltage of 20.00 kV and a working distance of 10 mm. To account for heterogeneity, several regions of each sample were examined. Quantification was performed using the AZtecEnergy software version 2.4 (Oxford Instruments) with standard ZAF correction. Results are expressed as weight percentages (W_t_, normalized to 100%) of major elements: silicon (Si), aluminum (Al), sodium (Na), calcium (Ca), potassium (K), magnesium (Mg), and iron (Fe).

#### 3.3.2. Exchangeable Cations and Cation Exchange Capacity

To extract exchangeable cations, 20 mg of gently ground, dried bentonite was mixed with 11 mL of 1 M NH_4_Cl in a centrifuge tube and shaken for 24 h. The sample was then centrifuged, and 10 mL of supernatant were collected. The residue was subsequently shaken with 10 mL of fresh 1 M NH_4_Cl for 2 h, centrifuged, and another 10 mL of supernatant were collected. This step was repeated once more. The collected supernatants were pooled, and the concentrations of elements (Si, Al, Na, Ca, Mg, K, and Fe) released by the NH_4_Cl treatment were determined by inductively coupled plasma–optical emission spectrometry (ICP-OES) using an ICPE-9800 system with an AS-10 autosampler (Shimadzu Corporation, Kyoto, Japan) in both axial and radial viewing modes. The instrument was operated at an RF power of 1.15 kW with plasma, auxiliary, and nebulizer gas flows set to 12, 0.5, and 0.5 L/min, respectively. Sample introduction was achieved using a concentric nebulizer coupled to a cyclonic spray chamber, with argon serving both as the plasma gas and for optical purging. Calibration curves for quantification were constructed based on the analysis of elemental standard solutions. The cation exchange capacity (CEC) of bentonite was calculated by summing the charges of all displaced cations, expressed in meq/100 g.

#### 3.3.3. Swelling Capacity

The swelling capacity of the bentonite samples was determined by the method described in the OIV Resolution OENO 441-2011 [14]. Two g of dried bentonite were dispersed into 100 mL of demineralized water in a graduated cylinder. The suspension was left to stand for 24 h, after which the swollen bentonite volume was measured. Results were expressed in mL/g of bentonite.

#### 3.3.4. Particle Size

Particle size distribution of the bentonite samples was determined by laser diffraction (static light scattering, SLS) using a Mastersizer 3000 instrument (Malvern Panalytical, Malvern, UK) fitted with a Hydro EV wet-dispersion unit. Scattering was recorded with both red and blue light sources, and the signals were combined to obtain volume-based size distributions. The initial suspension, prepared at 1 g/L bentonite in water, was allowed to stabilize overnight. For analysis, this suspension was diluted with deionized water to 0.1 g/L, subjected to 5 min of pre-sonication, and circulated through the measurement cell while maintaining an obscuration of approximately 5%. Measurements were carried out at 25 °C and processed using Mie theory (particle refractive index 1.473, absorption 0.01; dispersant refractive index 1.33). For each sample, ten consecutive runs were performed, with blanks subtracted and results averaged. Results are reported as volume-based distributions, where D10, D50, and D90 values denote the diameters below which 10%, 50%, and 90% of the sample volume comprises smaller particles, respectively. The D50 value corresponds to the median diameter of the distribution.

#### 3.3.5. External Specific Surface Area

The external specific surface area of the bentonite samples was calculated from the adsorption isotherm data using the Brunauer–Emmett–Teller (BET) method [69]. Before measurement, bentonites were gently ground and dried, and 0.25 mg of the sample was degassed under vacuum at 423 K for 3 h. Nitrogen adsorption–desorption isotherms were obtained at 77.35 K using an Autosorb iQ-MP surface area analyser (Quantachrome Instruments, Boynton Beach, FL, USA), using a previously determined fixed 52-point *p*/*p*^0^ table. All the measured isotherms were classified as type IV, displaying a hysteresis loop, a typical feature of mesoporous materials [35]. The hysteresis loop was visible in the range of 0.45–0.99 *p*/*p*^0^. Regarding the application of the multi-point BET calculations of external specific surface areas, the data in the range of 0.05–0.3 *p*/*p*^0^ was used. External specific surface area (E-SSA) was reported in m^2^/g of dry bentonite.

#### 3.3.6. Internal Specific Surface Area

The internal specific surface area of bentonite was determined by the methylene blue adsorption method described in the OIV Resolution OENO 441-2011 [14]. A suspension of 10 g bentonite in 200 mL deionized water was allowed to swell for 2 h, homogenized, and titrated with a 10 g/L methylene blue solution under constant stirring. After each addition (2 mL initially, then 1 mL, later 0.2–0.1 mL), adsorption was monitored by spot testing on filter paper. The endpoint was defined as the appearance of a persistent light blue ring around the deposit for 5 min. The required methylene blue volume (mL) was recorded, multiplied by 10, and internal specific surface area (I-SSA) was expressed in g/100 g.

#### 3.3.7. ζ-Potential

The electrokinetic *ζ*-potential (zeta potential) of bentonite samples was deter-mined by electrophoretic light scattering using a ZetaPlus Zeta Potential Analyzer (Brookhaven Instruments Corporation, Nashua, NH, USA) at room temperature. The initial suspension of bentonite in water at a mass concentration of 1 g/L was left overnight to stabilize. For measurement, the sample of the initial suspension was diluted in deionized water to 0.1 g/L. The suspension pH was adjusted to typical wine values in the range 3.0–3.6 by incremental additions of diluted HCl, and *ζ*-potential values were recorded at each pH. For each data point, at least three measurements were performed and averaged.

### 3.4. Batch-Scale Wine Clarification Trials

Each bentonite sample was suspended in deionized water following the producers’ instructions, thoroughly mixed, and left to stand overnight before application. In preliminary trials, to determine the minimum bentonite dose required for complete removal of PR proteins and wine stabilization, a range of doses was tested in increments of 10 g/hL for each bentonite. Wine aliquots were treated with varying amounts of bentonite, followed by assessment using the standard heat stability test, as previously described by Pocock et al. [70]. The test involved filtering a 20 mL wine sample through a 0.45 µm PTFE syringe filter, heating it at 80 °C for 2 h in a drying oven, cooling it at 4 °C for 2 h in a refrigerator, and then allowing it to stabilize at room temperature (24 °C). Haze was measured in nephelometric turbidity units (NTUs) using a nephelometric turbidity metre (Hanna Instruments HI 83749, Padova, Italy). The bentonite dose was considered sufficient for protein stabilization when the difference in haze between the heated/cooled and unheated samples was less than 2 NTU. The minimum effective dose of each bentonite, sufficient to achieve complete clarification and protein stabilization, was used in subsequent laboratory-scale wine batch clarification trials.

Each bentonite was tested in triplicate. The minimum effective dose of each bentonite was prepared as an aqueous suspension and added to 350 mL of wine in 1 L graduated glass cylinders. A control (wine without bentonite) was prepared in triplicate under identical conditions. Immediately after dosing, deionized water was added where needed to equalize dilution across treatments so that the total added-water volume was the same for all cylinders. Cylinders were mixed vigorously for 15 s and allowed to settle for 48 h at 20 °C. Clear supernatant wine aliquots were then withdrawn, filtered through Whatman 595 1/2 filter paper (Whatman GmbH, Dassel, Germany), and used for physico-chemical analyses. The remaining contents were left undisturbed for an additional 10 days, after which the sediment volume was recorded and reported as % of the total volume.

### 3.5. Chemical Analysis of Clarified Wines

#### 3.5.1. Elements

The elemental analysis of the treated and control wines was performed by ICP-OES (Shimadzu Corporation) after microwave digestion (Ethos UP, Millestone Srl, Milan, Italy). Microwave digestion was performed using 8 mL of HNO_3_ added to 0.2 mL of wine sample. The programme was set for a 25 min ramp to 200 °C and held for 15 min. The operating parameters were the same as described for the analysis of exchangeable cations (Section 3.3.2). Sample introduction was achieved using a concentric nebulizer coupled to a cyclonic spray chamber, with argon serving both as the plasma gas and for optical purging. Calibration curves were constructed for quantification.

#### 3.5.2. Phenolic Compounds

Phenolic compounds in treated wines were analyzed by high-performance liquid chromatography (HPLC) following the method detailed in Horvat et al. [71]. An Agilent Infinity 1260 system (Agilent Technologies, Santa Clara, CA, USA) was used, equipped with a G1311B quaternary pump, a G1329B autosampler, a G1316A column oven, and a G4212B DAD detector. Wine samples were filtered through 0.45 μm PTFE membranes, and 10 μL aliquots were injected onto a Poroshell 120 EC-C18 column (150 × 4.6 mm, 2.7 μm; Agilent) with a matching guard column (5 × 4.6 mm, 2.7 μm). The mobile phases were water/formic acid (99:1, *v*/*v*) (A) and acetonitrile (B), using a gradient programme as previously reported [70]. Column temperature was maintained at 26 °C. Detection was performed at 280 nm (for hydroxybenzoic acids, flavan-3-ols, stilbenes, taxifolin, and tyrosol) and 330 nm (for hydroxycinnamic acids), with full spectra recorded from 200 to 600 nm. Compounds were identified by matching retention times and UV spectra to pure standards. Calibration curves were constructed for quantification. For phenolic acids available only as qualitative standards (*cis*-caftaric, *cis*/*trans*-coutaric, and *cis*/*trans*-fertaric acids), semi-quantitative results were expressed in *trans*-caftaric acid equivalents, assuming similar detector response.

#### 3.5.3. Volatile Compounds

Prior to GC/MS analysis, volatile aroma compounds were extracted using HS-SPME based on Bubola et al. [72], with minor modifications. Wine samples were diluted 1:4 with deionized water and placed in 10 mL glass vials with 1 g of ammonium sulphate. A 50 μL internal standard mix (2-octanol, 1-nonanol, heptanoic acid) was added. A DVB/CAR/PDMS fibre was conditioned above the sample for 15 min at 40 °C, then exposed to headspace for 40 min at 40 °C with stirring (800 rpm). Volatiles were desorbed in a GC/MS injector at 248 °C for 10 min (3 min splitless). Analysis was performed on a Varian 3900 GC with a Saturn 2100T ion trap MS (Varian Inc., Harbor City, CA, USA), using a 60 m × 0.25 mm i.d. × 0.25 μm d.f. Rtx-WAX column (Restek, Bellefonte, PA, USA). The oven programme started at 40 °C, ramped at 2 °C/min to 240 °C, and held for 10 min. Helium was the carrier gas (1.2 mL/min). Mass spectra were acquired in EI mode (70 eV) over 30–350 *m*/*z*. Compounds were identified by comparing retention times and spectra with standards and the NIST05 library (reverse match factor > 750); lower matches were eventually manually verified by ion ratios. Linear retention indices (C10–C28 n-alkanes) were compared with literature for confirmation. Quantification was performed by calibration curves normalized to internal standards. Major volatiles were quantified using total ion current peak areas and minor ones by quantifier ion peak areas. Method validation details are reported in Bubola et al. [72]. For compounds without standards, semi-quantitative analysis was performed assuming similar detector responses.

### 3.6. Statistical Analysis

Data were analyzed by one-way ANOVA, and when significant, group means were compared using the least significant difference (LSD) post hoc test. To obtain the per-gram values, the measured changes in the concentrations of wine constituents were divided by the mass (in grams) of bentonite used in each treatment. Pearson correlation coefficients were calculated between the physico-chemical properties of bentonites and the concentrations of wine constituents, analyzed both as absolute values and as changes normalized per gram of bentonite. The significance threshold was set at *p* < 0.05 for all tests. To visualize sample relationships, hierarchical cluster analysis (HCA) was performed. All measurements were run in triplicate, except BET, which was measured in duplicate; for correlation analysis and HCA, the third BET replicate was imputed as the arithmetic mean of the two. ANOVA and correlation analyses were conducted in Statistica 13.2 (StatSoft Inc., Tulsa, OK, USA), and HCA in MetaboAnalyst 6.0 (www.metaboanalyst.ca; accessed 19 August 2025).

## 4. Conclusions

The results of this study demonstrated that the physico-chemical properties of bentonite strongly influence both protein adsorption and alterations of the wine matrix during clarification. Protein removal efficiency, expressed inversely as the dose required for complete stabilization, correlated positively with Na/Ca ratio, cation exchange capacity, swelling capacity, the magnitude of the negative *ζ*-potential, and internal specific surface area, and negatively with particle size and external specific surface area. Although these properties co-varied, the relationships were non-linear, indicating combined rather than singular effects. Overall, protein removal efficiency was most closely linked to the quantities and ratios of bentonite’s primary exchangeable cations, Na^+^ and Ca^2+^. Bentonite treatments significantly altered wine composition. Among the elements, Na and Ca showed the greatest increases in concentration, which corresponded closely to their contents in the bentonites. While effects on individual low-molecular-weight phenols were less clear and uniform, all bentonites removed a portion of total phenols. The levels of volatile compounds, especially esters, were also significantly reduced. When compared on a per-gram basis, bentonites with higher protein-removal efficiency generally exhibited stronger interactions with phenolic and volatile constituents, resulting in their greater removal. However, when evaluated at the full application doses required to achieve protein stability, these differences largely diminished or reversed, reflecting the compensatory effect of dosage. This highlights the importance of considering both the intrinsic reactivity of the clay material and its practical efficiency under application conditions when selecting an appropriate bentonite for targeted wine clarification. This study provided new insights into the mechanisms of bentonite–wine interactions and may contribute to more informed selection of bentonites in oenological practice. The wide variability of effects observed here and in previous studies underscores the complexity of these processes and the need for continued research.

## Figures and Tables

**Table 1:** Physico-chemical properties and performance of bentonites used for clarification of Malvazija istarska white wine.

Property	Bentonite							
	B1	B2	B3	B4	B5	B6	B7	B8
	Elemental Composition (W_t_/%)							
Si	73.7 ^a^	59.7 ^d^	70.3 ^b^	73.0 ^a^	60.0 ^d^	67.8 ^c^	60.8 ^d^	61.1 ^d^
Al	13.4 ^f^	23.1 ^a^	15.9 ^e^	13.9 ^f^	23.0 ^a^	17.3 ^d^	21.5 ^b^	19.9 ^c^
Fe	3.08 ^d^	5.11 ^bc^	4.37 ^c^	2.55 ^d^	5.06 ^c^	4.72 ^c^	6.07 ^b^	8.43 ^a^
Na	1.88 ^d^	3.39 ^a^	2.80 ^c^	3.47 ^a^	3.42 ^a^	2.81 ^c^	3.09 ^b^	0.89 ^e^
Ca	2.94 ^b^	2.30 ^c^	2.37 ^c^	2.25 ^c^	3.16 ^b^	2.45 ^c^	2.39 ^c^	3.87 ^a^
Na/Ca	0.64 ^c^	1.52 ^a^	1.18 ^b^	1.57 ^a^	1.09 ^b^	1.15 ^b^	1.29 ^ab^	0.23 ^d^
Mg	2.65 ^d^	2.65 ^d^	1.84 ^e^	3.17 ^b^	2.77 ^cd^	3.10 ^bc^	3.08 ^bc^	3.85 ^a^
K	2.37 ^b^	2.89 ^ab^	2.44 ^ab^	1.68 ^c^	2.60 ^ab^	1.60 ^c^	3.01 ^a^	1.71 ^c^
	NH_4_Cl Extract Composition (meq/100 g)							
Si	0.37 ^d^	0.63 ^d^	3.75 ^a^	2.54 ^b^	0.54 ^d^	1.74 ^c^	0.57 ^d^	0.69 ^d^
Al	0.14 ^ab^	0.15 ^ab^	0.03 ^c^	0.05 ^c^	0.15 ^ab^	0.05 ^c^	0.12 ^b^	0.20 ^a^
Na	29.7 ^e^	49.0 ^cd^	42.5 ^d^	47.7 ^cd^	71.2 ^a^	64.0 ^ab^	56.4 ^bc^	14.0 ^f^
Ca	14.9 ^b^	11.3 ^c^	11.4 ^c^	14.5 ^b^	12.0 ^c^	11.7 ^c^	12.3 ^c^	18.6 ^a^
Na/Ca	2.00 ^e^	4.33 ^bc^	3.75 ^cd^	3.27 ^d^	5.96 ^a^	5.44 ^a^	4.57 ^b^	0.75 ^f^
Mg	5.80 ^d^	7.30 ^bc^	7.88 ^b^	6.22 ^cd^	7.44 ^bc^	10.61 ^a^	7.94 ^b^	8.12 ^b^
K	4.58 ^abc^	5.71 ^a^	2.99 ^de^	4.71 ^ab^	3.47 ^cde^	3.86 ^bcd^	3.83 ^bcd^	2.60 ^e^
Mn	0.39 ^a^	0.00 ^d^	0.01 ^d^	0.21 ^b^	0.01 ^d^	0.00 ^d^	0.00 ^d^	0.07 ^c^
	Cation Exchange Capacity (meq/100 g)							
CEC	55.4 ^de^	73.3 ^bc^	64.7 ^cd^	73.4 ^bc^	94.1 ^a^	90.1 ^a^	80.5 ^ab^	43.4 ^e^
	Swelling Capacity (mL/g)							
Swelling Capacity	5.17 ^d^	8.33 ^b^	5.67 ^d^	8.67 ^b^	11.0 ^a^	7.00 ^c^	7.00 ^c^	3.83 ^e^
	Particle Size ^1^ (μm)							
D10	0.45 ^b^	0.38 ^f^	0.45 ^b^	0.40 ^d^	0.37 ^f^	0.42 ^c^	0.39 ^e^	1.89 ^a^
D50	0.59 ^b^	0.52 ^e^	0.60 ^b^	0.55 ^d^	0.52 ^e^	0.57 ^c^	0.54 ^d^	2.76 ^a^
D90	1.08 ^b^	0.84 ^f^	0.98 ^c^	0.91 ^d^	0.84 ^f^	0.93 ^d^	0.87 ^e^	5.06 ^a^
	Specific Surface Area							
E-SSA ^2^ (m^2^/g)	59.2 ^b^	57.0 ^c^	57.1 ^c^	36.7 ^f^	23.6 ^g^	55.4 ^d^	50.0 ^e^	66.9 ^a^
I-SSA ^3^ (g/100 g)	21.0 ^f^	43.8 ^a^	29.1 ^e^	35.7 ^bc^	42.0 ^a^	31.8 ^d^	37.8 ^b^	34.5 ^c^
	Surface Charge (mV)							
*ζ*-Potential	−36.4 ^a^	−32.5 ^a^	−35.7 ^a^	−51.1 ^b^	−47.9 ^b^	−48.9 ^b^	−42.4 ^ab^	−33.3 ^a^
	Bentonite Performance							
Dose (g/hL)	380 ^b^	180 ^f^	300 ^c^	160 ^h^	200 ^e^	170 ^g^	220 ^d^	650 ^a^
Sediment (%)	14.5 ^d^	18.6 ^b^	13.1 ^f^	29.3 ^a^	14.2 ^e^	17.1 ^c^	14.2 ^e^	1.50 ^g^

^1^ D10, D50, and D90 denote the particle diameters at which 10%, 50%, and 90% of the sample volume consist of smaller particles, respectively. D50 represents the median diameter of the distribution. ^2^ External specific surface area. ^3^ Internal specific surface area. Different lowercase superscript letters in a row represent statistically significant differences between the concentrations in Malvazija istarska wine after clarification by different bentonites, as determined by one-way ANOVA and LSD test at *p* < 0.05.

**Table 2:** Changes (%) in the concentration of elements in Malvazija istarska wine after clarification with different bentonites.

Element	γ ^1^	Bentonite/Change in Concentration							
		B1	B2	B3	B4	B5	B6	B7	B8
K	390 ^ab^	−1.54 ^ab^	**+4.53 ^a^**	+1.88 ^ab^	+0.51 ^ab^	+2.05 ^ab^	−1.37 ^ab^	+0.43 ^ab^	−5.47 ^b^
P	144 ^ab^	+0.93 ^ab^	−3.02 ^ab^	−6.73 ^b^	−3.02 ^ab^	−1.86 ^ab^	−7.42 ^b^	+3.25 ^a^	−3.02 ^ab^
S	125 ^ab^	−2.92 ^ab^	−5.04 ^ab^	−8.22 ^b^	−6.63 ^b^	−3.18 ^ab^	−7.96 ^b^	+2.12 ^a^	−1.06 ^ab^
Mg	117 ^cd^	−5.11 ^d^	−2.27 ^cd^	−0.57 ^cd^	−0.85 ^cd^	+3.98 ^abc^	+1.99 ^bcd^	**+10.2 ^ab^**	**+11.1 ^a^**
Ca	71.2 ^c^	+41.3 ^b^	+2.34 ^bc^	+6.69 ^bc^	+6.69 ^bc^	+16.0 ^bc^	+29.6 ^bc^	+25.1 ^bc^	**+83.5 ^a^**
Na	9.03 ^f^	**+211 ^c^**	**+202 ^cd^**	**+301 ^a^**	**+213 ^c^**	**+254 ^b^**	**+174 ^de^**	**+270 ^b^**	**+165 ^e^**
Al	3.67 ^d^	**+27.2 ^b^**	+27.9 ^b^	**+22.1 ^bc^**	**+16.0 ^bcd^**	+12.8 ^bcd^	+4.27 ^cd^	**+54.5 ^a^**	+12.9 ^bcd^
Fe	3.10 ^ab^	+6.14 ^ab^	+4.20 ^ab^	−0.11 ^ab^	+1.18 ^ab^	+15.2 ^a^	−4.31 ^b^	+12.5 ^a^	+15.6 ^a^
Mn	1.23 ^c^	**+75.7 ^a^**	+0.54 ^c^	+1.08 ^c^	**+19.2 ^b^**	+4.32 ^c^	−0.54 ^c^	**+8.38 ^bc^**	**+80.5 ^a^**
Zn	0.72 ^ab^	+6.48 ^a^	−0.93 ^ab^	**−22.7 ^c^**	−15.3 ^bc^	−14.8 ^bc^	**−24.5 ^c^**	−11.1 ^abc^	−11.1 ^abc^
Co	0.05 ^ab^	−7.14 ^ab^	+14.3 ^a^	−7.14 ^ab^	0.00 ^ab^	−14.3 ^b^	0.00 ^ab^	−7.14 ^ab^	+14.3 ^a^

^1^ Concentration (mg/L) of elements in non-clarified Malvazija istarska control C wine. Different lowercase superscript letters in a row represent statistically significant differences between the concentrations in Malvazija istarska wine after clarification with different bentonites, as determined by one-way ANOVA and LSD test at *p* < 0.05. Bold values represent concentrations that are significantly different from those found in control C wine, as determined by *t*-test at *p* < 0.05. Plus (+) represents an increase and minus (−) a decrease in concentration.

**Table 3:** Changes (%) in the concentration of phenolic compounds in Malvazija istarska wine after clarification with different bentonites.

Phenols	γ ^1^	Bentonite/Change in Concentration (%)							
		B1	B2	B3	B4	B5	B6	B7	B8
	Hydroxybenzoic acids								
Gallic acid	1.39 ^ab^	+1.01 ^ab^	+1.85 ^ab^	+0.70 ^ab^	−3.69 ^b^	+3.39 ^ab^	−1.71 ^ab^	−4.35 ^b^	+6.15 ^a^
Protocatechuic acid	0.72 ^bc^	**+9.42 ^a^**	+2.36 ^abc^	+1.90 ^bc^	+3.85 ^ab^	−2.37 ^bc^	−2.30 ^bc^	−3.86 ^c^	−1.05 ^bc^
*p*-Hydroxybenzoic acid	0.43 ^bc^	+9.24 ^a^	+2.41 ^b^	+3.00 ^b^	−1.90 ^bc^	−0.77 ^bc^	−4.88 ^c^	−5.68 ^c^	−4.04 ^c^
2.5-Dihydroxybenzoic acid	0.66 ^abc^	+4.12 ^a^	+0.97 ^ab^	−2.59 ^bcd^	−4.55 ^bcd^	−2.28 ^bcd^	**−7.61 ^d^**	−5.27 ^cd^	−6.22 ^d^
	Hydroxycinnamic acids								
*cis*-Caftaric acid	0.19 ^bcd^	−25.4 ^e^	−3.33 ^cd^	−16.4 ^de^	+18.6 ^ab^	+19.6 ^ab^	+16.1 ^abc^	+16.7 ^abc^	+21.6 ^a^
*trans*-Caftaric acid	3.32 ^c^	**+9.03 ^a^**	**+7.86 ^a^**	+8.77 ^a^	+4.82 ^ab^	**+6.94 ^ab^**	+2.79 ^bc^	**+4.53 ^abc^**	**+6.94 ^ab^**
*cis*-Coutaric acid	0.97 ^bc^	**+5.05 ^a^**	**+5.13 ^a^**	+5.29 ^a^	+1.82 ^abc^	+2.65 ^abc^	−0.65 ^c^	+0.55 ^bc^	+3.66 ^ab^
*trans*-Coutaric acid	0.14 ^c^	**+18.9 ^ab^**	**+16.7 ^ab^**	**+21.6 ^a^**	**+17.5 ^ab^**	**+13.7 ^b^**	**+14.5 ^b^**	**+18.4 ^ab^**	**+21.6 ^a^**
*cis*-Fertaric acid	0.23 ^bcd^	**+8.17 ^a^**	+4.88 ^abc^	+5.17 ^ab^	+3.01 ^abcd^	−0.15 ^cd^	−1.21 ^d^	+0.95 ^bcd^	−0.84 ^d^
*trans*-Fertaric acid	1.91 ^c^	**+5.31 ^a^**	**+5.77 ^a^**	+5.99 ^a^	+2.34 ^abc^	+3.73 ^abc^	+0.19 ^bc^	+1.02 ^bc^	+4.20 ^ab^
Caffeic acid	5.74 ^ab^	−4.12 ^bc^	+0.49 ^ab^	−5.97 ^c^	+3.88 ^a^	+3.67 ^a^	+0.14 ^ab^	+1.30 ^a^	+3.04 ^a^
*p*-Coumaric acid	1.48 ^abc^	−3.25 ^bc^	+0.93 ^ab^	−4.65 ^c^	+3.75 ^a^	+3.88 ^a^	+0.20 ^ab^	+1.19 ^ab^	+3.36 ^a^
Ferulic acid	1.12 ^abc^	−0.38 ^abc^	+1.95 ^a^	−2.33 ^bc^	+1.28 ^ab^	+0.97 ^abc^	−2.42 ^c^	−1.28 ^abc^	−0.19 ^abc^
	Flavan-3-ols								
Epicatechin	0.96 ^ab^	+3.41 ^a^	−3.90 ^ab^	−2.98 ^ab^	−3.75 ^ab^	−7.58 ^ab^	−3.27 ^ab^	−0.29 ^ab^	**−13.4 ^b^**
Procyanidin B1	1.82 ^ab^	−25.8 ^c^	+2.98 ^ab^	−8.74 ^bc^	+20.6 ^a^	+20.4 ^a^	+16.3 ^a^	+19.7 ^a^	+19.8 ^a^
Procyanidin B2	0.78 ^de^	−6.71 ^e^	+2.12 ^cde^	+3.50 ^bcde^	+14.5 ^ab^	+16.0 ^a^	+15.0 ^ab^	+12.7 ^abc^	+7.91 ^abcd^
	Stilbenes								
*cis*-Piceid	0.04	+2.46	+3.96	−2.79	−1.63	+0.05	−1.32	−0.01	+1.92
*trans*-Piceid	0.07 ^a^	−20.0 ^cd^	−16.0 ^bcd^	−21.1 ^d^	−5.76 ^ab^	−10.8 ^bc^	−12.7 ^bcd^	−13.5 ^bcd^	−12.4 ^bcd^
	Miscellaneous								
Taxifolin	0.21 ^abc^	−24.7 ^d^	−2.66 ^bc^	−17.3 ^cd^	+17.0 ^a^	+15.9 ^ab^	+12.6 ^ab^	+13.3 ^ab^	+17.1 ^a^
Catechin + tyrosol	21.3 ^bc^	+1.35 ^ab^	+3.65 ^a^	+0.31 ^abc^	+1.47 ^ab^	+1.39 ^ab^	−2.23 ^c^	−1.11 ^bc^	+0.81 ^abc^
Total phenols	239 ^a^	−11.0 ^bc^	−7.04 ^b^	−9.63 ^bc^	−8.09 ^bc^	−13.0 ^c^	−10.5 ^bc^	−10.4 ^bc^	−12.2 ^bc^

^1^ Concentration (mg/L) of phenolic compounds in non-clarified Malvazija istarska control C wine. Different lowercase superscript letters in a row represent statistically significant differences between the concentrations in Malvazija istarska wine after clarification with different bentonites, as determined by one-way ANOVA and LSD test at *p* < 0.05. Bold values represent concentrations that are significantly different from those found in control C wine, as determined by *t*-test at *p* < 0.05. Plus (+) represents an increase and minus (−) a decrease in concentration.

**Table 4:** Changes (%) in the concentration of volatile compounds in Malvazija istarska wine after clarification with different bentonites.

Volatile Compound	γ ^1^	Bentonite/Change in Concentration (%)							
		B1	B2	B3	B4	B5	B6	B7	B8
	Monoterpenoids								
Linalool	0.027	+0.90	+5.54	+3.05	+3.72	+1.94	+2.51	+1.18	**+1.67**
Geraniol	0.004 ^ab^	+0.29 ^ab^	−8.85 ^ab^	+3.04 ^a^	−9.08 ^ab^	−3.51 ^ab^	−2.93 ^ab^	−15.47 ^b^	**−4.15 ^ab^**
6-Methyl-5-hepten-2-ol	0.044 ^bc^	+13.0 ^ab^	+14.8 ^ab^	+18.9 ^a^	+16.0 ^ab^	−5.10 ^c^	+8.11 ^abc^	+15.9 ^ab^	+5.62 ^abc^
	C_13_-Norisoprenoids								
β-Damascenone	0.002 ^a^	−15.3 ^bc^	−15.9 ^bc^	−12.9 ^bc^	−13.0 ^bc^	−14.8 ^bc^	−10.7 ^b^	−16.6 ^bc^	−18.1 ^c^
	Alcohols								
1-Hexanol	1.11	−0.12	+2.27	−0.30	+2.11	−0.77	+2.49	+7.04	−2.30
*trans*-3-Hexen-1-ol	0.093	+0.29	+2.97	−1.65	+3.90	−3.68	+3.47	**−1.19**	**−0.52**
*cis*-3-Hexen-1-ol	0.054 ^bc^	+7.39 ^abc^	+2.31 ^abc^	+18.6 ^a^	+8.33 ^abc^	+5.56 ^abc^	+7.38 ^abc^	+16.5 ^ab^	**−8.40 ^c^**
2-Phenylethanol	32.2 ^ab^	−1.33 ^ab^	−14.2 ^d^	**+3.41 ^a^**	−8.81 ^bcd^	−4.23 ^abc^	**−8.29 ^bcd^**	**−13.1 ^cd^**	−8.51 ^bcd^
	Fatty Acids								
Hexanoic acid	4.42 ^a^	−0.85 ^a^	−24.8 ^b^	−5.08 ^a^	−21.1 ^b^	−25.2 ^b^	**−25.3 ^b^**	**+6.70 ^a^**	**−22.5 ^b^**
Octanoic acid	4.27 ^bc^	+4.05 ^b^	−2.47 ^bc^	+15.5 ^a^	−4.62 ^bc^	+0.14 ^bc^	−1.32 b^c^	+6.56 ^ab^	−9.04 ^c^
	Ethyl Esters								
Ethyl propanoate	0.030	−1.63	+0.95	+2.58	+4.19	+1.62	+7.55	+2.29	+0.44
Ethyl isobutyrate	0.018 ^a^	−11.82 ^c^	−8.72 ^abc^	−10.81 ^bc^	−8.71 ^abc^	−5.97 ^abc^	−2.48 ^ab^	−0.81 ^a^	−8.58 ^abc^
Ethyl butyrate	0.25 ^ab^	**−12.6 ^b^**	**−6.38 ^ab^**	**−6.67 ^ab^**	**−1.16 ^ab^**	**−3.17 ^ab^**	**+2.21 ^a^**	**−2.42 ^ab^**	**−6.99 ^ab^**
Ethyl 2-methylbutyrate	0.011 ^a^	−20.9 ^c^	−13.3 ^bc^	−17.0 ^bc^	−11.0 ^bc^	−11.9 ^bc^	−10.2 ^ab^	−10.6 ^bc^	−18.8 ^bc^
Ethyl 3-methylbutyrate	0.015 ^a^	−12.3 ^ab^	−26.7 ^b^	−18.1 ^ab^	−20.7 ^ab^	−26.7 ^b^	**−1.21 ^a^**	**−0.15 ^a^**	**−23.8 ^ab^**
Ethyl hexanoate	0.70 ^a^	−21.1 ^d^	−13.0 ^bcd^	−16.5 ^cd^	−7.94 ^abc^	−16.5 ^cd^	**−6.77 ^ab^**	**−15.7 ^bcd^**	−14.4 ^bcd^
Ethyl octanoate	1.70 ^a^	**−12.7 ^b^**	**−4.46 ^ab^**	**−10.8 ^b^**	**−8.96 ^ab^**	**−8.69 ^ab^**	**−9.65 ^ab^**	**−10.3 ^ab^**	**−10.7 ^b^**
Ethyl nonanoate	0.022 ^a^	−21.6 ^bc^	−29.1 ^c^	−18.2 ^b^	−19.3 ^b^	−22.8 ^bc^	**−19.2 ^b^**	**−22.7 ^bc^**	**−16.2 ^b^**
Ethyl 2-furoate	0.002 ^a^	−4.67 ^ab^	−16.6 ^c^	**+0.21 ^a^**	−10.8 ^bc^	−8.00 ^abc^	**−9.92 ^abc^**	**−10.3 ^bc^**	−11.3 ^bc^
Ethyl decanoate	1.84 ^a^	−41.7 ^de^	−14.8 ^b^	−37.0 ^cde^	−25.1 ^bc^	−32.6 ^cd^	−14.5 ^b^	−48.3 ^e^	−36.3 ^cde^
	Acetate Esters								
Methyl acetate	0.015 ^bc^	−8.78 ^c^	−5.54 ^c^	−10.4 ^c^	−8.83 ^c^	−4.88 ^bc^	+26.5 ^a^	−3.54 ^bc^	+8.63 ^b^
Propyl acetate	0.018 ^ab^	−5.66 ^bc^	−18.9 ^d^	−7.39 ^bc^	−11.9 ^cd^	−5.74 ^bc^	**−14.5 ^cd^**	**+5.83 ^a^**	**+9.83 ^a^**
Isobutyl acetate	0.046 ^a^	−8.61 ^bc^	−9.48 ^bc^	−9.74 ^c^	−7.21 ^abc^	−2.19 ^abc^	−1.05 ^ab^	−4.65 ^abc^	**−9.41 ^bc^**
Butyl acetate	0.016 ^a^	−37.9 ^bc^	−36.9 ^bc^	−36.3 ^bc^	−30.5 ^bc^	−36.7 ^bc^	−42.0 ^c^	−28.9 ^b^	**−39.8 ^bc^**
Isoamyl acetate	2.12 ^a^	**−12.2 ^b^**	**−9.18 ^ab^**	**−7.22 ^ab^**	**−12.4 ^b^**	**−10.6 ^ab^**	**−6.82 ^ab^**	**−5.25 ^ab^**	**−11.3 ^ab^**
Hexyl acetate	0.036 ^a^	−23.4 ^c^	−10.5 ^abc^	−11.0 ^abc^	−10.2 ^abc^	−9.75 ^abc^	−1.95 ^a^	−22.8 ^bc^	−4.88 ^ab^
*cis*-3-Hexen-1-yl acetate	0.029 ^a^	−18.6 ^c^	−16.9 ^bc^	−6.53 ^ab^	−17.5 ^c^	−11.3 ^bc^	−6.81 ^ab^	**−14.0 ^bc^**	**−8.34 ^abc^**
*trans*-3-Hexen-1-yl acet.	0.016 ^a^	−14.3 ^ab^	−14.9 ^b^	−0.16 ^a^	−12.2 ^ab^	−5.93 ^ab^	−3.54 ^ab^	−9.04 ^ab^	−3.51 ^ab^
2-Phenethyl acetate	0.12 ^ab^	−2.56 ^abc^	−10.5 ^bcd^	+3.30 ^a^	−13.2 ^cd^	−7.97 ^bcd^	−13.2 ^cd^	−15.2 ^d^	−9.28 ^bcd^
	Other Esters								
Methyl decanoate	0.002 ^a^	**−45.1 ^ef^**	**−21.8 ^b^**	**−34.4 ^cde^**	**−29.8 ^bcd^**	**−32.5 ^bcd^**	**−22.0 ^bc^**	**−40.6 ^def^**	**−48.2 ^f^**
Diethyl succinate	2.72 ^ab^	−0.18 ^ab^	−11.5 ^c^	+10.1 ^a^	−2.50 ^bc^	−2.84 ^bc^	−4.64 ^bc^	**−6.84 ^bc^**	**−38.2 ^d^**
Isoamyl decanoate	0.026 ^ab^	**+11.0 ^ab^**	**−6.04 ^ab^**	**+15.9 ^a^**	**−6.56 ^ab^**	**+0.17 ^ab^**	**+0.75 ^ab^**	**−13.2 ^b^**	**−2.43 ^ab^**

^1^ Concentration (mg/L) of volatile compounds in non-clarified Malvazija istarska control C wine. Different lowercase superscript letters in a row represent statistically significant differences between the concentrations in Malvazija istarska wine after clarification with different bentonites, as determined by one-way ANOVA and LSD test at *p* < 0.05. Bold values represent concentrations that are significantly different from those found in control C wine, as determined by *t*-test at *p* < 0.05. Plus (+) represents an increase and minus (−) a decrease in concentration.

## Data Availability

Dataset available on request from the authors.

## Supplementary Materials

The following supporting information can be downloaded at https://www.mdpi.com/article/10.3390/molecules30204117/s1: Table S1: Pairwise Pearson’s correlation coefficients (*r*) among physico-chemical properties across eight bentonites (*n* = 8). Significant correlations (*p* < 0.05) are shown in red; Table S2: Pairwise Pearson correlation coefficients (*r*) between physico-chemical properties of eight bentonites and (i) element concentrations in the corresponding clarified wines and (ii) changes in element concentrations normalized per gram of bentonite (*n* = 8). Significant correlations (*p* < 0.05) are shown in red; Table S3: Pairwise Pearson correlation coefficients (*r*) between physico-chemical properties of eight bentonites and (i) concentrations of phenolic compounds in the corresponding clarified wines and (ii) changes in phenolic compound concentrations normalized per gram of bentonite (*n* = 8). Significant correlations (*p* < 0.05) are shown in red; Table S4: Pairwise Pearson correlation coefficients (*r*) between physico-chemical properties of eight bentonites and concentrations of volatile compounds in the corresponding clarified wines (*n* = 8). Significant correlations (*p* < 0.05) are shown in red; Table S5: Pairwise Pearson correlation coefficients (*r*) between physico-chemical properties of eight bentonites and changes in volatile compound concentrations normalized per gram of bentonite (*n* = 8). Significant correlations (*p* < 0.05) are shown in red.
