# Supplementary material for: Bentonite-Clarified White Wine: Linking Clay Physico-Chemical Properties to Protein Removal Efficiency and Wine Matrix Alterations
Source: Molecules. 2025 Oct 17;30(20):4117. doi: 10.3390/molecules30204117 (PMC12565947; doi:10.3390/molecules30204117)
Supplement: Supplementary file 1 [file molecules-30-04117-s001.zip › molecules-3928219-supplementary.pdf]

Supplementary Materials

Table S1. Pairwise Pearson's correlation coefficients (r) among physico-chemical properties across eight bentonites ($n = 8$). Significant correlations ($p < 0.05$) are shown in red.

Bentonite properties	W_t(Si) (%)	W_t(Al) (%)	W_t(Na) (%)	W_t(Ca) (%)	W_t(Na) / W_t(Ca)	W_t(Mg) (%)	W_t(K) (%)	W_t(Fe) (%)	Si (meq/100g)	Al (meq/100g)	Na (meq/100g)	Ca (meq/100g)	Na/Ca (meq/100g)	Mg (meq/100g)	K (meq/100g)	Mn (meq/100g)	CEC (meq/100g)	SC (mL/g)	D10-SLS (μm)	D50-SLS (μm)	D90-SLS (μm)	E-SSA-BET (m^2/g)	I-SSA-MBT (g/100g)	ζ-Potential (mV)	Dose (g/hL)	Sediment (%)
**Near-Surface Elemental Composition**																										
**W_t(Si) (%)**		-0.97	-0.06	-0.30	0.04	-0.29	-0.44	-0.75	0.54	-0.57	-0.21	0.07	-0.27	-0.28	0.09	0.69	-0.22	-0.25	-0.27	-0.28	-0.27	0.09	-0.78	-0.17	-0.08	0.44
**W_t(Al) (%)**	-0.97		0.21	0.17	0.09	0.16	0.49	0.61	-0.51	0.48	0.32	-0.21	0.40	0.24	-0.01	-0.71	0.32	0.39	0.10	0.12	0.10	-0.19	0.81	0.13	-0.08	-0.33
**W_t(Na) (%)**	-0.06	0.21		-0.71	0.91	-0.43	0.33	-0.50	0.23	-0.44	0.80	-0.73	0.80	-0.03	0.43	-0.31	0.75	0.82	-0.83	-0.83	-0.84	-0.69	0.51	-0.44	-0.95	0.77
**W_t(Ca) (%)**	-0.30	0.17	-0.71		-0.88	0.49	-0.25	0.56	-0.45	0.52	-0.48	0.57	-0.49	-0.06	-0.47	0.08	-0.45	-0.31	0.75	0.75	0.76	0.15	-0.07	0.28	0.76	-0.72
**W_t(Na)/W_t(Ca)**	0.04	0.09	0.91	-0.88		-0.40	0.27	-0.48	0.35	-0.45	0.65	-0.64	0.65	0.01	0.49	-0.24	0.61	0.60	-0.74	-0.74	-0.75	-0.45	0.42	-0.38	-0.87	0.79
**W_t(Mg) (%)**	-0.29	0.16	-0.43	0.49	-0.40		-0.43	0.45	-0.46	0.38	-0.26	0.61	-0.34	0.16	-0.11	0.03	-0.17	-0.15	0.62	0.63	0.62	0.09	0.19	-0.10	0.39	-0.22
**W_t(K) (%)**	-0.44	0.49	0.33	-0.25	0.27	-0.43		0.09	-0.28	0.32	0.29	-0.36	0.33	-0.21	0.17	-0.20	0.24	-0.25	-0.38	-0.37	-0.38	-0.12	0.28	0.30	-0.24	-0.04
**W_t(Fe) (%)**	-0.75	0.61	-0.50	0.56	-0.48	0.45	0.09		-0.35	0.59	-0.24	0.33	-0.19	0.42	-0.43	-0.52	-0.20	-0.33	0.74	0.74	0.73	0.36	0.35	0.27	0.60	-0.80
**NH_4Cl Extract and Cation Exchange Capacity**																										
**Si (meq/100g)**	0.54	-0.51	0.23	-0.45	0.35	-0.46	-0.28	-0.35		-0.74	0.07	-0.22	0.08	0.16	-0.18	-0.13	0.04	-0.08	-0.19	-0.20	-0.21	0.00	-0.22	-0.19	-0.21	0.32
**Al (meq/100g)**	-0.57	0.48	-0.44	0.52	-0.45	0.38	0.32	0.59	-0.74		-0.30	0.46	-0.30	-0.18	-0.01	0.10	-0.26	-0.08	0.49	0.50	0.50	0.11	0.22	0.27	0.51	-0.53
**Na (meq/100g)**	-0.21	0.32	0.80	-0.48	0.65	-0.26	0.29	-0.24	0.07	-0.30		-0.65	0.97	0.38	0.22	-0.42	0.99	0.77	-0.71	-0.70	-0.71	-0.68	0.44	-0.60	-0.83	0.47
**Ca (meq/100g)**	0.07	-0.21	-0.73	0.57	-0.64	0.61	-0.36	0.33	-0.22	0.46	-0.65		-0.78	-0.17	-0.22	0.47	-0.55	-0.52	0.78	0.77	0.78	0.32	-0.26	0.12	0.77	-0.41
**Na/Ca (meq/100g)**	-0.27	0.40	0.80	-0.49	0.65	-0.34	0.33	-0.19	0.08	-0.30	0.97	-0.78		0.42	0.18	-0.54	0.94	0.75	-0.71	-0.70	-0.71	-0.61	0.47	-0.48	-0.82	0.40
**Mg (meq/100g)**	-0.28	0.24	-0.03	-0.06	0.01	0.16	-0.21	0.42	0.16	-0.18	0.38	-0.17	0.42		-0.23	-0.62	0.44	-0.08	0.11	0.12	0.10	0.20	0.12	-0.28	-0.07	-0.25
**K (meq/100g)**	0.09	-0.01	0.43	-0.47	0.49	-0.11	0.17	-0.43	-0.18	-0.01	0.22	-0.22	0.18	-0.23		0.26	0.24	0.35	-0.49	-0.49	-0.48	-0.08	0.19	-0.12	-0.50	0.60
**Mn (meq/100g)**	0.69	-0.71	-0.31	0.08	-0.24	0.03	-0.20	-0.52	-0.13	0.10	-0.42	0.47	-0.54	-0.62	0.26		-0.40	-0.27	-0.02	-0.03	-0.01	0.09	-0.66	-0.02	0.22	0.22
**CEC (meq/100g)**	-0.22	0.32	0.75	-0.45	0.61	-0.17	0.24	-0.20	0.04	-0.26	0.99	-0.55	0.94	0.44	0.24	-0.40		0.74	-0.64	-0.64	-0.65	-0.65	0.44	-0.64	-0.78	0.45
**Swelling Capacity**																										
**SC (mL/g)**	-0.25	0.39	0.82	-0.31	0.60	-0.15	-0.25	-0.33	-0.08	-0.08	0.77	-0.52	0.75	-0.08	0.35	-0.27	0.74		-0.61	-0.60	-0.61	-0.89	0.64	-0.48	-0.76	0.58
**Particle Size**																										
**D10-SLS (μm)**	-0.27	0.10	-0.83	0.75	-0.74	0.62	-0.38	0.74	-0.19	0.49	-0.71	0.78	-0.71	0.11	-0.49	-0.02	-0.64	-0.61		1.00	1.00	0.50	-0.05	0.35	0.91	-0.74
**D50-SLS (μm)**	-0.28	0.12	-0.83	0.75	-0.74	0.63	-0.37	0.74	-0.20	0.50	-0.70	0.77	-0.70	0.12	-0.49	-0.03	-0.64	-0.60	1.00		1.00	0.49	-0.03	0.35	0.90	-0.74
**D90-SLS (μm)**	-0.27	0.10	-0.84	0.76	-0.75	0.62	-0.38	0.73	-0.21	0.50	-0.71	0.78	-0.71	0.10	-0.48	-0.01	-0.65	-0.61	1.00	1.00		0.49	-0.05	0.35	0.91	-0.74
**Specific Surface Area**																										
**E-SSA-BET (m^2/g)**	0.09	-0.19	-0.69	0.15	-0.45	0.09	-0.12	0.36	0.00	0.11	-0.68	0.32	-0.61	0.20	-0.08	0.09	-0.65	-0.89	0.50	0.49	0.49		-0.45	0.60	0.60	-0.51
**I-SSA-MBT (g/100g)**	-0.78	0.81	0.51	-0.07	0.42	0.19	0.28	0.35	-0.22	0.22	0.44	-0.26	0.47	0.12	0.19	-0.66	0.44	0.64	-0.05	-0.03	-0.05	-0.45		-0.14	-0.35	0.13
**Surface Charge**																										
**ζ-Potential (mV)**	-0.17	0.13	-0.44	0.28	-0.38	-0.10	0.30	0.27	-0.19	0.27	-0.60	0.12	-0.48	-0.28	-0.12	-0.02	-0.64	-0.48	0.35	0.35	0.35	0.60	-0.14		0.49	-0.48
**Bentonite performance**																										
**Dose (g/hL)**	-0.08	-0.08	-0.95	0.76	-0.87	0.39	-0.24	0.60	-0.21	0.51	-0.83	0.77	-0.82	-0.07	-0.50	0.22	-0.78	-0.76	0.91	0.90	0.91	0.60	-0.35	0.49		-0.82
**Sediment (%)**	0.44	-0.33	0.77	-0.72	0.79	-0.22	-0.04	-0.80	0.32	-0.53	0.47	-0.41	0.40	-0.25	0.60	0.22	0.45	0.58	-0.74	-0.74	-0.74	-0.51	0.13	-0.48	-0.82	

Table S2. Pairwise Pearson correlation coefficients (*r*) between physico-chemical properties of eight bentonites and (i) element concentrations in the corresponding clarified wines and (ii) changes in element concentrations normalized per gram of bentonite ($n = 8$). Significant correlations ($p < 0.05$) are shown in red.

Bentonite properties	W_t(Si) (%)	W_t(Al) (%)	W_t(Na) (%)	W_t(Ca) (%)	W_t(Na) / W_t(Ca)	W_t(Mg) (%)	W_t(K) (%)	W_t(Fe) (%)	Si (meq/100g)	Al (meq/100g)	Na (meq/100g)	Ca (meq/100g)	Na/Ca (meq/100g)	Mg (meq/100g)	K (meq/100g)	Mn (meq/100g)	CEC (meq/100g)	SC (mL/g)	D10-SLS (µm)	D50-SLS (µm)	D90-SLS (µm)	E-SSA-BET (m^2/g)	I-SSA-MBT (g/100g)	ζ-Potential (mV)	Dose (g/hL)	Sediment (%)
**Elements in Wine (mg/L)**																										
K (mg/L)	-0.04	0.15	0.38	-0.38	0.35	-0.25	0.19	-0.25	0.09	-0.07	0.25	-0.38	0.31	-0.03	0.19	-0.17	0.21	0.40	-0.39	-0.39	-0.40	-0.23	0.18	-0.12	-0.39	0.28
P (mg/L)	-0.10	0.11	-0.08	0.00	-0.10	0.19	0.21	0.01	**-0.41**	0.38	-0.14	0.11	-0.14	-0.30	0.02	0.19	-0.15	0.08	-0.04	-0.03	-0.03	-0.08	-0.02	0.00	0.05	-0.03
S (mg/L)	-0.29	0.27	-0.23	0.17	-0.25	0.34	0.16	0.29	**-0.45**	**0.48**	-0.17	0.22	-0.16	-0.09	-0.17	0.01	-0.16	-0.04	0.20	0.20	0.20	0.02	0.06	0.03	0.22	-0.28
Mg (mg/L)	**-0.48**	0.40	-0.28	0.30	-0.31	**0.52**	-0.06	**0.60**	-0.21	0.33	-0.08	0.31	-0.09	0.29	**-0.49**	-0.39	-0.05	-0.09	**0.46**	**0.46**	**0.46**	0.03	0.26	-0.06	0.31	**-0.45**
Ca (mg/L)	-0.13	-0.02	**-0.74**	**0.59**	**-0.70**	**0.57**	-0.23	**0.50**	-0.30	**0.44**	-0.38	**0.71**	**-0.46**	0.23	-0.37	0.16	-0.29	**-0.53**	**0.68**	**0.68**	**0.68**	0.40	-0.26	0.14	**0.69**	**-0.60**
Na (mg/L)	0.04	0.05	**0.42**	-0.35	0.33	**-0.62**	**0.48**	-0.23	0.37	-0.27	0.32	**-0.45**	0.35	-0.14	-0.18	-0.21	0.25	0.23	**-0.47**	**-0.47**	**-0.48**	-0.33	-0.01	-0.06	-0.31	0.10
Al (mg/L)	-0.13	0.18	0.17	-0.30	0.26	-0.21	**0.45**	-0.01	-0.22	0.06	-0.05	-0.17	-0.01	-0.25	0.15	0.00	-0.09	-0.08	-0.21	-0.21	-0.21	0.09	0.03	0.18	-0.09	0.00
Fe (mg/L)	**-0.45**	0.39	-0.20	0.34	-0.22	0.16	0.32	**0.49**	**-0.42**	**0.68**	-0.03	0.26	-0.04	-0.04	-0.18	-0.05	-0.01	0.03	0.32	0.33	0.33	-0.13	0.19	-0.04	0.33	-0.39
Mn (mg/L)	0.20	-0.32	**-0.86**	**0.62**	**-0.78**	**0.44**	-0.26	0.23	-0.38	**0.54**	**-0.79**	**0.86**	**-0.86**	-0.34	-0.19	**0.66**	**-0.74**	**-0.64**	**0.68**	**0.67**	**0.69**	**0.46**	**-0.52**	0.33	**0.83**	**-0.50**
Zn (mg/L)	-0.03	0.04	-0.22	0.09	-0.24	0.06	0.40	-0.09	**-0.48**	**0.51**	-0.28	0.34	-0.33	**-0.50**	0.33	**0.48**	-0.26	-0.08	0.02	0.01	0.03	0.17	-0.09	0.30	0.17	-0.04
Co (mg/L)	-0.18	0.11	-0.19	0.02	-0.02	0.16	-0.03	0.30	-0.05	0.19	-0.20	0.28	-0.21	0.17	0.15	-0.07	-0.14	-0.23	0.35	0.35	0.35	0.36	0.19	0.26	0.23	-0.11
**Change in Element Concentrations per Gram of Bentonite**																										
K (Δ/g)	0.26	-0.21	0.09	-0.09	0.14	-0.09	**-0.48**	-0.21	0.31	-0.39	0.02	0.00	0.00	0.10	-0.14	0.06	0.02	-0.10	-0.03	-0.03	-0.04	-0.04	-0.11	-0.28	-0.04	0.08
P (Δ/g)	-0.10	0.12	-0.29	0.24	-0.24	-0.01	-0.20	0.15	-0.17	0.13	-0.28	0.12	-0.23	0.09	-0.05	0.03	-0.27	-0.31	0.25	0.25	0.26	0.37	-0.09	0.30	0.32	-0.38
S (Δ/g)	0.01	-0.03	**-0.47**	0.32	-0.39	0.04	-0.28	0.18	-0.16	0.13	**-0.42**	0.26	-0.38	0.09	-0.13	0.15	-0.40	-0.52	0.36	0.36	0.36	**0.50**	-0.29	0.31	**0.48**	**-0.48**
Mg (Δ/g)	0.08	-0.11	-0.21	0.13	-0.13	0.30	**-0.51**	0.12	-0.02	-0.14	-0.04	0.21	-0.07	0.35	-0.29	0.02	0.00	-0.29	0.23	0.23	0.23	0.11	-0.22	-0.23	0.19	-0.21
Ca (Δ/g)	-0.33	0.39	0.30	-0.22	0.33	-0.03	0.04	0.07	-0.21	-0.03	0.17	-0.22	0.21	0.15	**0.43**	-0.30	0.18	0.16	-0.13	-0.12	-0.13	0.08	0.37	0.03	-0.25	0.12
Na (Δ/g)	0.02	0.06	**0.70**	**-0.50**	**0.71**	0.11	-0.30	-0.32	0.35	**-0.49**	**0.57**	-0.34	**0.53**	0.23	0.29	-0.33	**0.58**	**0.63**	-0.37	-0.37	-0.38	**-0.52**	**0.52**	**-0.59**	**-0.70**	**0.73**
Al (Δ/g)	-0.12	0.14	0.28	-0.19	0.34	0.06	-0.11	-0.02	0.33	-0.11	0.09	-0.13	0.13	0.02	0.27	-0.28	0.10	0.32	0.01	0.01	0.00	-0.14	**0.52**	-0.08	-0.22	0.32
Fe (Δ/g)	0.02	-0.01	-0.03	-0.03	-0.04	0.05	-0.37	0.03	0.14	-0.20	0.23	-0.06	0.23	**0.59**	0.09	-0.21	0.28	-0.03	0.01	0.01	0.01	0.18	0.02	-0.20	-0.09	-0.01
Mn (Δ/g)	**-0.48**	**0.56**	0.37	-0.23	**0.41**	-0.15	0.36	0.05	-0.28	0.23	0.17	-0.33	0.26	-0.04	**0.57**	-0.29	0.16	0.35	-0.19	-0.18	-0.18	0.02	**0.58**	0.29	-0.29	0.16
Zn (Δ/g)	-0.12	0.11	-0.28	0.12	-0.17	-0.29	0.16	0.17	-0.10	0.24	-0.30	0.04	-0.22	0.00	0.21	0.04	-0.29	-0.35	0.18	0.18	0.18	**0.48**	-0.15	**0.53**	0.34	-0.38
Co (Δ/g)	0.02	-0.02	-0.14	0.11	-0.21	-0.13	0.23	0.02	0.06	0.09	-0.14	-0.01	-0.09	-0.18	-0.13	-0.03	-0.17	-0.04	0.03	0.03	0.03	0.01	-0.15	0.34	0.16	-0.19

Table S3. Pairwise Pearson correlation coefficients (*r*) between physico-chemical properties of eight bentonites and (i) concentrations of phenolic compounds in the corresponding clarified wines and (ii) changes in phenolic compound concentrations normalized per gram of bentonite (n = 8). Significant correlations ($p < 0.05$) are shown in red.

Bentonite properties	W_t(Si) (%)	W_t(Al) (%)	W_t(Na) (%)	W_t(Ca) (%)	W_t(Na) / W_t(Ca)	W_t(Mg) (%)	W_t(K) (%)	W_t(Fe) (%)	Si (meq/100g)	Al (meq/100g)	Na (meq/100g)	Ca (meq/100g)	Na/Ca (meq/100g)	Mg (meq/100g)	K (meq/100g)	Mn (meq/100g)	CEC (meq/100g)	SC (mL/g)	D10-SLS (µm)	D50-SLS (µm)	D90-SLS (µm)	E-SSA-BET (m^2/g)	I-SSA-MBT (g/100g)	ζ-Potential (mV)	Dose (g/hL)	Sediment (%)
**Phenols in Wine (mg/L)**																										
**Gallic acid**	-0.24	0.18	-0.37	0.47	-0.41	0.13	0.02	0.33	-0.18	0.31	-0.21	0.27	-0.18	0.12	-0.24	-0.07	-0.19	-0.12	0.41	0.41	0.41	0.15	-0.03	0.35	0.43	-0.45
**Protocatechuic acid**	0.49	-0.50	-0.14	-0.09	-0.08	-0.20	0.06	-0.41	0.05	0.00	-0.33	0.20	-0.38	-0.50	0.27	0.64	-0.33	-0.20	-0.12	-0.13	-0.11	0.16	-0.41	0.20	0.08	0.22
***p*-Hydroxybenzoic acid**	0.43	-0.38	-0.12	-0.05	-0.15	-0.51	0.24	-0.45	0.02	0.01	-0.24	0.01	-0.24	-0.52	0.25	0.56	-0.27	-0.11	-0.24	-0.25	-0.23	0.14	-0.44	0.34	0.06	0.07
**2,5-DiOHbenzoic acid**	0.19	-0.12	0.00	-0.11	-0.02	-0.38	0.39	-0.35	-0.20	0.17	-0.15	0.02	-0.17	-0.55	0.33	0.47	-0.18	0.03	-0.26	-0.27	-0.25	0.04	-0.20	0.34	-0.02	0.09
***cis*-Caftaric acid**	-0.46	0.41	0.13	0.13	0.13	0.63	-0.25	0.40	-0.16	0.11	0.29	0.15	0.24	0.38	-0.21	-0.44	0.34	0.30	0.29	0.30	0.28	-0.38	0.55	-0.37	-0.05	0.00
***trans*-Caftaric acid**	0.08	-0.07	-0.20	0.12	-0.21	-0.28	0.27	-0.06	-0.01	0.14	-0.30	0.13	-0.28	-0.33	-0.07	0.21	-0.33	-0.15	0.07	0.06	0.07	0.14	-0.21	0.40	0.26	-0.21
***cis*-Coutaric acid**	0.09	-0.07	-0.21	0.08	-0.18	-0.29	0.25	-0.06	0.04	0.14	-0.38	0.16	-0.35	-0.37	0.03	0.22	-0.40	-0.19	0.11	0.10	0.11	0.23	-0.17	0.47	0.28	-0.18
***trans*-Coutaric acid**	0.13	-0.23	-0.44	0.21	-0.35	-0.03	0.10	0.22	0.18	0.00	-0.51	0.31	-0.50	-0.11	-0.38	0.09	-0.51	-0.57	0.38	0.37	0.38	0.46	-0.35	0.48	0.49	-0.32
***cis*-Fertaric acid**	0.43	-0.39	0.02	-0.22	0.05	-0.45	0.31	-0.48	0.10	-0.17	-0.23	-0.01	-0.26	-0.53	0.25	0.49	-0.27	-0.16	-0.30	-0.31	-0.29	0.18	-0.38	0.39	-0.04	0.21
***trans*-Fertaric acid**	0.05	-0.03	-0.17	0.08	-0.16	-0.29	0.22	-0.06	0.06	0.12	-0.32	0.13	-0.29	-0.32	0.02	0.16	-0.34	-0.14	0.09	0.09	0.09	0.17	-0.13	0.40	0.25	-0.18
**Caffeic acid**	-0.40	0.37	0.15	0.10	0.15	0.60	-0.11	0.24	-0.31	0.24	0.21	0.25	0.13	0.10	-0.02	-0.18	0.26	0.38	0.22	0.23	0.22	-0.41	0.54	-0.24	-0.06	0.11
***p*-Coumaric acid**	-0.40	0.37	0.13	0.13	0.13	0.58	-0.11	0.25	-0.30	0.26	0.18	0.26	0.11	0.09	-0.03	-0.18	0.23	0.37	0.24	0.25	0.24	-0.40	0.53	-0.21	-0.03	0.08
**Ferulic acid**	-0.16	0.19	0.17	-0.03	0.17	0.18	0.15	-0.09	-0.26	0.20	0.01	0.17	-0.04	-0.32	0.26	0.12	0.02	0.31	-0.01	0.00	0.00	-0.26	0.34	0.11	-0.08	0.21
**Epicatechin**	0.24	-0.22	0.13	-0.47	0.24	-0.33	0.44	-0.26	0.04	-0.04	0.20	-0.10	0.14	-0.10	0.19	0.24	0.20	0.01	-0.37	-0.38	-0.37	0.02	-0.25	-0.02	-0.24	0.24
**Procyanidin B1**	-0.49	0.45	0.23	0.03	0.24	0.53	-0.17	0.39	-0.08	0.03	0.35	0.04	0.31	0.38	-0.16	-0.53	0.39	0.33	0.21	0.22	0.21	-0.39	0.62	-0.37	-0.14	0.05
**Procyanidin B2**	-0.26	0.27	0.36	-0.11	0.32	0.33	-0.17	0.13	0.11	-0.21	0.52	-0.16	0.49	0.44	-0.21	-0.49	0.54	0.45	-0.04	-0.03	-0.04	-0.52	0.44	-0.52	-0.32	0.19
***cis*-Piceid**	-0.23	0.20	-0.18	0.07	-0.11	0.12	0.25	0.20	-0.45	0.41	-0.19	0.19	-0.17	-0.12	0.17	0.14	-0.17	-0.08	0.15	0.15	0.16	0.19	0.10	0.36	0.18	-0.16
***trans*-Piceid**	-0.18	0.16	0.31	-0.06	0.34	0.53	-0.28	0.05	-0.09	0.01	0.31	0.17	0.20	0.10	0.10	-0.11	0.36	0.43	0.08	0.09	0.08	-0.51	0.48	-0.49	-0.23	0.37
**Taxifolin**	-0.45	0.41	0.17	0.11	0.17	0.63	-0.25	0.37	-0.16	0.09	0.29	0.13	0.25	0.36	-0.16	-0.42	0.34	0.32	0.26	0.27	0.26	-0.39	0.57	-0.37	-0.09	0.04
**Catechin (+ tyrosol)**	-0.09	0.12	0.08	-0.01	0.07	-0.04	0.22	-0.12	-0.15	0.17	-0.16	0.15	-0.18	-0.42	0.29	0.17	-0.16	0.15	0.00	0.01	0.01	-0.07	0.21	0.31	0.03	0.11
**Total phenols**	0.06	-0.05	0.30	-0.38	0.43	-0.20	0.14	-0.19	0.16	-0.17	0.04	-0.22	0.04	-0.17	0.42	-0.02	0.02	0.01	-0.22	-0.22	-0.22	0.09	0.13	0.23	-0.27	0.37
**Change in Phenolic Compound Concentrations per Gram of Bentonite**																										
**Gallic acid**	-0.24	0.23	-0.17	0.32	-0.25	-0.05	0.18	0.19	-0.20	0.20	-0.03	0.02	0.02	0.07	-0.16	-0.13	-0.03	0.03	0.17	0.17	0.17	0.04	0.01	0.40	0.22	-0.33
**Protocatechuic acid**	0.42	-0.43	-0.07	-0.12	0.02	-0.07	0.01	-0.38	0.13	-0.03	-0.32	0.21	-0.38	-0.50	0.26	0.52	-0.33	-0.11	-0.06	-0.07	-0.06	0.10	-0.24	0.18	0.04	0.29
***p*-Hydroxybenzoic acid**	0.23	-0.20	-0.21	0.13	-0.25	-0.40	0.26	-0.24	-0.03	0.13	-0.36	0.10	-0.35	-0.54	0.15	0.42	-0.40	-0.14	-0.03	-0.04	-0.03	0.20	-0.27	0.49	0.22	-0.08
**2,5-DiOHbenzoic acid**	-0.03	0.05	-0.24	0.17	-0.24	-0.22	0.38	-0.03	-0.26	0.35	-0.39	0.18	-0.37	-0.54	0.11	0.32	-0.42	-0.15	0.08	0.08	0.09	0.20	-0.11	0.55	0.30	-0.23
***cis*-Caftaric acid**	-0.21	0.21	0.36	-0.11	0.35	0.46	-0.24	0.06	-0.05	-0.09	0.49	-0.03	0.42	0.32	-0.07	-0.28	0.53	0.46	-0.04	-0.03	-0.04	-0.55	0.42	-0.54	-0.33	0.29
***trans*-Caftaric acid**	-0.05	0.16	0.49	-0.33	0.43	-0.31	0.37	-0.35	0.04	-0.14	0.27	-0.37	0.30	-0.28	0.28	-0.08	0.22	0.48	-0.44	-0.43	-0.44	-0.32	0.29	0.09	-0.43	0.38
***cis*-Coutaric acid**	-0.04	0.10	0.18	-0.16	0.18	-0.31	0.37	-0.21	0.03	0.03	-0.08	-0.14	-0.05	-0.41	0.25	0.04	-0.13	0.18	-0.17	-0.17	-0.17	-0.03	0.15	0.37	-0.10	0.15
***trans*-Coutaric acid**	0.10	-0.02	0.74	-0.63	0.73	-0.16	0.17	-0.45	0.28	-0.52	0.54	-0.50	0.52	0.02	0.32	-0.22	0.51	0.57	-0.60	-0.60	-0.61	-0.38	0.31	-0.25	-0.76	0.76
***cis*-Fertaric acid**	0.26	-0.21	0.15	-0.24	0.18	-0.28	0.29	-0.40	0.10	-0.19	-0.17	-0.03	-0.20	-0.51	0.27	0.29	-0.21	-0.01	-0.24	-0.25	-0.24	0.10	-0.10	0.38	-0.12	0.31
***trans*-Fertaric acid**	-0.07	0.15	0.28	-0.19	0.25	-0.32	0.33	-0.24	0.05	-0.03	0.03	-0.21	0.06	-0.34	0.28	-0.02	-0.02	0.28	-0.24	-0.24	-0.24	-0.12	0.23	0.26	-0.19	0.20
**Caffeic acid**	-0.21	0.21	0.31	-0.06	0.32	0.46	-0.08	-0.03	-0.20	0.09	0.30	0.11	0.22	-0.03	0.06	-0.06	0.33	0.47	0.00	0.01	0.00	-0.54	0.43	-0.27	-0.23	0.32
***p*-Coumaric acid**	-0.21	0.22	0.33	-0.06	0.33	0.43	-0.06	-0.04	-0.19	0.08	0.30	0.09	0.22	-0.05	0.07	-0.07	0.33	0.50	-0.02	-0.01	-0.01	-0.55	0.45	-0.26	-0.24	0.32
**Ferulic acid**	-0.13	0.16	0.18	-0.02	0.19	0.15	0.17	-0.14	-0.21	0.17	-0.05	0.13	-0.09	-0.44	0.24	0.14	-0.06	0.30	-0.01	0.00	0.00	-0.26	0.32	0.22	-0.06	0.23
**Epicatechin**	0.13	-0.17	-0.15	-0.23	0.00	-0.15	0.37	0.02	0.00	0.12	-0.04	0.12	-0.09	-0.04	-0.03	0.19	-0.02	-0.22	-0.03	-0.04	-0.03	0.21	-0.28	0.14	0.08	-0.04
**Procyanidin B1**	-0.25	0.27	0.47	-0.22	0.47	0.39	-0.16	0.05	0.00	-0.14	0.55	-0.12	0.49	0.31	0.03	-0.36	0.58	0.52	-0.11	-0.10	-0.11	-0.57	0.51	-0.54	-0.42	0.36
**Procyanidin B2**	-0.06	0.10	0.53	-0.30	0.48	0.25	-0.21	-0.15	0.15	-0.34	0.65	-0.27	0.60	0.37	-0.03	-0.33	0.67	0.58	-0.28	-0.27	-0.29	-0.63	0.36	-0.63	-0.55	0.46
***cis*-Piceid**	-0.32	0.33	-0.05	-0.01	0.02	0.05	0.39	0.15	-0.45	0.36	-0.13	-0.02	-0.06	-0.17	0.21	0.01	-0.14	0.00	0.05	0.05	0.05	0.18	0.18	0.51	0.08	-0.13
***trans*-Piceid**	0.06	-0.18	-0.46	0.45	-0.35	0.51	-0.35	0.26	-0.11	0.31	-0.41	0.72	-0.52	-0.18	-0.30	0.33	-0.36	-0.28	0.62	0.62	0.62	-0.02	-0.15	-0.03	0.57	-0.25
**Taxifolin**	-0.21	0.20	0.36	-0.11	0.36	0.48	-0.25	0.05	-0.06	-0.09	0.46	-0.02	0.39	0.28	-0.03	-0.26	0.50	0.46	-0.03	-0.02	-0.03	-0.54	0.43	-0.51	-0.32	0.31
**Catechin (+ tyrosol)**	-0.12	0.17	0.20	-0.06	0.19	-0.03	0.25	-0.18	-0.13	0.12	-0.10	0.03	-0.10	-0.48	0.34	0.10	-0.12	0.26	-0.07	-0.07	-0.07	-0.15	0.30	0.32	-0.08	0.22
**Total phenols**	0.05	-0.16	-0.58	0.29	-0.40	0.00	0.08	0.28	-0.03	0.33	-0.70	0.47	-0.69	-0.26	-0.05	0.26	-0.68	-0.68	0.51	0.50	0.51	0.63	-0.34	0.68	0.65	-0.44

Table S4. Pairwise Pearson correlation coefficients (*r*) between physico-chemical properties of eight bentonites and concentrations of volatile compounds in the corresponding clarified wines ($n = 8$). Significant correlations ($p < 0.05$) are shown in red.

Bentonite properties	W_t(Si) (%)	W_t(Al) (%)	W_t(Na) (%)	W_t(Ca) (%)	W_t(Na) / W_t(Ca)	W_t(Mg) (%)	W_t(K) (%)	W_t(Fe) (%)	Si (meq/100g)	Al (meq/100g)	Na (meq/100g)	Ca (meq/100g)	Na/Ca (meq/100g)	Mg (meq/100g)	K (meq/100g)	Mn (meq/100g)	CEC (meq/100g)	SC (mL/g)	D10-SLS (μm)	D50-SLS (μm)	D90-SLS (μm)	E-SSA-BET (m^2/g)	I-SSA-MBT (g/100g)	ζ-Potential (mV)	Dose (g/hL)	Sediment (%)
**Volatile Compounds in Wine (mg/L)**																										
**6-Methyl-5-hepten-2-ol**	0.31	-0.29	0.08	-0.42	0.24	-0.23	0.15	-0.29	0.31	-0.21	-0.22	-0.05	-0.22	-0.23	0.26	0.13	-0.24	-0.23	-0.17	-0.17	-0.17	0.32	-0.25	0.27	-0.10	0.24
**Linalool**	-0.03	0.05	0.12	-0.11	0.12	0.02	0.00	-0.10	0.07	-0.13	0.07	0.04	0.04	-0.06	0.23	-0.05	0.09	0.12	-0.07	-0.07	-0.07	0.00	0.16	0.24	-0.12	0.14
**Geraniol**	0.30	-0.25	-0.21	0.07	-0.19	-0.22	-0.30	-0.16	0.24	-0.08	-0.20	0.05	-0.17	-0.06	-0.18	0.19	-0.22	-0.18	0.06	0.05	0.06	0.12	-0.34	-0.12	0.18	-0.16
**β-Damascenone**	0.32	-0.28	0.21	-0.23	0.18	-0.08	-0.37	-0.34	0.32	-0.55	0.25	-0.29	0.27	0.19	-0.02	-0.04	0.24	0.13	-0.30	-0.30	-0.30	-0.13	-0.11	-0.15	-0.32	0.30
**1-Hexanol**	-0.05	0.07	0.27	-0.30	0.31	0.08	0.13	-0.13	-0.05	-0.18	0.20	-0.19	0.18	0.06	0.18	-0.13	0.20	0.10	-0.25	-0.25	-0.25	-0.04	0.11	0.20	-0.29	0.21
***trans*-3-Hexen-1-ol**	0.19	-0.19	0.05	-0.20	0.16	0.16	-0.20	-0.23	0.05	-0.26	-0.01	0.00	-0.05	-0.05	0.21	0.07	-0.01	-0.03	-0.07	-0.07	-0.06	0.13	-0.02	0.28	-0.15	0.29
***cis*-3-Hexen-1-ol**	0.24	-0.19	0.37	-0.44	0.37	-0.38	0.22	-0.34	0.34	-0.43	0.32	-0.37	0.30	-0.03	0.06	-0.04	0.28	0.11	-0.49	-0.49	-0.49	-0.15	-0.11	0.09	-0.39	0.27
**2-Phenylethanol**	0.47	-0.42	-0.16	0.07	-0.21	-0.52	-0.15	-0.27	0.40	-0.32	-0.11	0.00	-0.11	-0.15	-0.34	0.25	-0.15	-0.20	-0.06	-0.07	-0.06	-0.01	-0.50	-0.09	0.17	-0.14
**Hexanoic acid**	0.23	-0.22	-0.12	-0.25	-0.03	-0.24	0.41	-0.14	0.01	-0.02	-0.12	0.01	-0.15	-0.24	-0.05	0.26	-0.15	-0.34	-0.19	-0.20	-0.19	0.21	-0.47	0.20	0.07	-0.10
**Octanoic acid**	0.25	-0.16	0.17	-0.39	0.19	-0.68	0.29	-0.26	0.36	-0.35	0.17	-0.39	0.21	-0.06	-0.05	-0.05	0.11	-0.06	-0.40	-0.41	-0.41	0.02	-0.29	0.05	-0.19	0.01
**Ethyl propanoate**	0.09	-0.06	0.14	-0.17	0.17	0.08	-0.28	-0.13	0.14	-0.37	0.15	-0.17	0.15	0.17	-0.26	-0.18	0.12	0.05	-0.10	-0.09	-0.10	-0.07	-0.03	-0.10	-0.19	0.15
**Ethyl isobutyrate**	-0.33	0.31	0.22	-0.04	0.16	0.30	0.00	0.20	-0.19	-0.07	0.39	-0.20	0.37	0.36	-0.18	-0.38	0.39	0.16	-0.11	-0.10	-0.11	-0.15	0.29	-0.12	-0.25	0.01
**Ethyl butyrate**	-0.10	0.09	0.28	-0.13	0.24	0.26	-0.15	-0.03	0.12	-0.29	0.38	-0.17	0.35	0.33	-0.14	-0.33	0.39	0.26	-0.13	-0.12	-0.13	-0.23	0.24	-0.07	-0.32	0.22
**Ethyl 2-methylbutyrate**	-0.18	0.22	0.50	-0.29	0.46	0.18	-0.03	-0.12	0.04	-0.24	0.45	-0.35	0.46	0.21	0.03	-0.36	0.44	0.45	-0.31	-0.30	-0.31	-0.37	0.40	-0.13	-0.50	0.36
**Ethyl 3-methylbutyrate**	0.14	-0.17	-0.04	-0.20	0.01	0.19	0.01	-0.05	0.03	-0.18	0.21	-0.07	0.17	0.40	0.03	-0.01	0.25	-0.11	-0.17	-0.17	-0.17	0.15	-0.25	-0.07	-0.13	0.03
**Ethyl hexanoate**	0.01	-0.02	0.29	-0.21	0.36	0.23	-0.38	-0.03	0.24	-0.40	0.28	-0.04	0.23	0.33	0.09	-0.20	0.32	0.11	-0.04	-0.03	-0.04	-0.09	0.28	-0.39	-0.29	0.35
**Ethyl octanoate**	-0.26	0.29	0.23	-0.11	0.21	0.03	0.09	0.05	-0.07	-0.08	0.21	-0.15	0.22	-0.01	0.30	-0.21	0.22	0.22	-0.11	-0.10	-0.10	-0.10	0.43	0.01	-0.22	0.16
**Ethyl nonanoate**	0.23	-0.33	-0.39	0.36	-0.41	0.21	-0.44	0.17	0.26	-0.13	-0.28	0.18	-0.26	0.13	-0.42	0.06	-0.28	-0.32	0.34	0.34	0.34	0.11	-0.31	-0.25	0.34	-0.21
**Ethyl 2-furoate**	0.43	-0.41	-0.14	0.02	-0.21	-0.46	-0.07	-0.21	0.40	-0.35	-0.07	-0.04	-0.08	-0.12	-0.38	0.20	-0.12	-0.23	-0.11	-0.12	-0.12	0.01	-0.45	-0.12	0.11	-0.12
**Ethyl decanoate**	-0.03	0.06	0.31	-0.14	0.31	0.08	-0.36	-0.13	0.13	-0.33	0.28	-0.31	0.32	0.34	0.38	-0.24	0.30	0.30	-0.16	-0.15	-0.16	-0.04	0.32	-0.20	-0.37	0.37
**Methyl acetate**	-0.15	0.11	-0.24	0.23	-0.27	0.49	-0.43	0.26	-0.13	-0.16	0.07	0.10	0.08	0.62	-0.16	-0.30	0.13	-0.17	0.27	0.27	0.26	0.25	-0.02	0.02	0.08	-0.23
**Propyl acetate**	-0.26	0.14	-0.53	0.45	-0.49	0.34	0.02	0.60	-0.26	0.51	-0.39	0.51	-0.42	-0.02	-0.50	0.00	-0.37	-0.41	0.58	0.58	0.58	0.18	-0.12	0.15	0.63	-0.62
**Isobutyl acetate**	-0.12	0.14	0.22	-0.06	0.12	0.22	-0.11	-0.04	-0.14	-0.14	0.43	-0.22	0.42	0.27	-0.19	-0.20	0.42	0.31	-0.21	-0.21	-0.21	-0.33	0.14	-0.20	-0.31	0.11
**Butyl acetate**	0.02	-0.02	0.32	-0.32	0.37	-0.12	0.29	-0.12	0.07	-0.04	0.18	-0.01	0.07	-0.29	0.07	0.04	0.17	0.18	-0.22	-0.22	-0.22	-0.26	0.16	-0.05	-0.23	0.30
**Isoamyl acetate**	-0.12	0.11	0.07	-0.15	0.08	-0.03	0.07	0.12	0.05	-0.14	0.12	-0.30	0.20	0.22	-0.20	-0.32	0.09	0.01	-0.12	-0.12	-0.12	0.07	0.07	0.20	-0.14	-0.05
**Hexyl acetate**	-0.10	0.09	-0.03	0.19	-0.06	0.23	-0.42	0.15	0.20	-0.22	0.08	0.08	0.08	0.33	-0.11	-0.26	0.12	0.03	0.22	0.23	0.22	0.01	0.24	-0.13	0.04	-0.05
***cis*-3-Hexen-1-yl acetate**	-0.12	0.10	-0.19	0.23	-0.26	-0.02	-0.28	0.32	0.24	-0.24	-0.01	-0.07	0.07	0.39	-0.53	-0.42	-0.02	-0.17	0.23	0.23	0.23	0.13	0.01	0.01	0.17	-0.36
***trans*-3-Hexen-1-yl acetate**	-0.06	0.04	-0.17	0.19	-0.23	0.06	-0.17	0.22	0.29	-0.25	0.04	0.09	0.03	0.34	-0.44	-0.30	0.06	-0.16	0.21	0.21	0.20	0.07	0.00	-0.01	0.17	-0.31
**2-Phenethyl acetate**	0.30	-0.25	-0.18	0.08	-0.20	-0.54	0.04	-0.17	0.29	-0.22	-0.25	-0.04	-0.20	-0.24	-0.24	0.17	-0.30	-0.29	-0.01	-0.02	-0.01	0.17	-0.40	0.19	0.22	-0.21
**Methyl decanoate**	-0.04	0.12	0.61	-0.46	0.59	-0.19	-0.09	-0.25	0.24	-0.48	0.56	-0.58	0.61	0.33	0.43	-0.35	0.55	0.47	-0.49	-0.48	-0.49	-0.25	0.41	-0.34	-0.66	0.52
**Diethyl succinate**	0.51	-0.36	0.59	-0.66	0.54	-0.78	0.22	-0.73	0.48	-0.62	0.48	-0.61	0.48	-0.16	0.19	0.13	0.41	0.30	-0.83	-0.83	-0.83	-0.36	-0.24	-0.32	-0.65	0.55
**Isoamyl decanoate**	0.36	-0.31	-0.18	0.01	-0.19	-0.40	-0.09	-0.20	0.27	-0.23	-0.14	0.03	-0.13	-0.10	-0.15	0.22	-0.16	-0.23	-0.04	-0.04	-0.04	0.14	-0.41	-0.05	0.14	-0.13

Table S5. Pairwise Pearson correlation coefficients (r) between physico-chemical properties of eight bentonites and changes in volatile compound concentrations normalized per gram of bentonite (n = 8). Significant correlations ($p < 0.05$) are shown in red.

Bentonite properties	W_t(Si) (%)	W_t(Al) (%)	W_t(Na) (%)	W_t(Ca) (%)	W_t(Na) / W_t(Ca)	W_t(Mg) (%)	W_t(K) (%)	W_t(Fe) (%)	Si (meq/100g)	Al (meq/100g)	Na (meq/100g)	Ca (meq/100g)	Na/Ca (meq/100g)	Mg (meq/100g)	K (meq/100g)	Mn (meq/100g)	CEC (meq/100g)	SC (mL/g)	D10-SLS (μm)	D50-SLS (μm)	D90-SLS (μm)	E-SSA-BET (m^2/g)	I-SSA-MBT (g/100g)	ζ-Potential (mV)	Dose (g/hL)	Sediment (%)
**Change in Volatile Compound Concentrations per Gram of Bentonite**																										
**6-Methyl-5-hepten-2-ol**	0.24	-0.21	0.30	-0.53	0.45	-0.15	0.10	-0.32	0.29	-0.26	-0.03	-0.14	-0.05	-0.15	0.42	0.04	-0.04	0.01	-0.27	-0.27	-0.28	0.14	-0.02	0.10	-0.31	0.46
**Linalool**	-0.02	0.07	0.28	-0.19	0.25	0.03	-0.03	-0.22	0.07	-0.27	0.17	-0.08	0.14	-0.04	0.40	-0.10	0.19	0.26	-0.19	-0.18	-0.19	-0.09	0.24	0.19	-0.29	0.32
**Geraniol**	0.21	-0.18	-0.30	0.16	-0.28	-0.15	-0.30	0.00	0.16	0.02	-0.26	0.12	-0.21	0.00	-0.25	0.16	-0.26	-0.26	0.17	0.16	0.17	0.17	-0.30	-0.11	0.28	-0.29
**β-Damascenone**	0.23	-0.32	-0.71	0.49	-0.69	0.17	-0.39	0.20	0.06	0.00	-0.55	0.40	-0.52	0.06	-0.51	0.21	-0.54	-0.64	0.51	0.50	0.51	0.46	-0.55	0.37	0.66	-0.56
**1-Hexanol**	-0.05	0.06	0.20	-0.25	0.26	0.15	0.07	-0.09	-0.05	-0.15	0.16	-0.12	0.13	0.11	0.15	-0.13	0.17	0.07	-0.16	-0.16	-0.16	0.00	0.09	0.21	-0.22	0.17
***trans*-3-Hexen-1-ol**	0.22	-0.22	0.07	-0.25	0.19	0.19	-0.23	-0.26	0.10	-0.31	-0.01	0.02	-0.05	0.04	0.22	0.05	0.01	-0.01	-0.07	-0.07	-0.07	0.13	-0.04	0.23	-0.17	0.33
***cis*-3-Hexen-1-ol**	0.17	-0.14	0.37	-0.42	0.38	-0.15	0.11	-0.29	0.30	-0.44	0.36	-0.27	0.31	0.10	0.07	-0.12	0.35	0.15	-0.40	-0.40	-0.40	-0.19	-0.03	0.01	-0.38	0.30
**2-Phenylethanol**	0.31	-0.32	-0.43	0.35	-0.48	-0.30	-0.18	0.02	0.23	-0.08	-0.32	0.23	-0.32	-0.13	-0.50	0.25	-0.34	-0.40	0.24	0.23	0.24	0.13	-0.50	0.02	0.46	-0.42
**Hexanoic acid**	0.08	-0.15	-0.47	0.12	-0.37	-0.05	0.30	0.22	-0.08	0.22	-0.42	0.26	-0.42	-0.19	-0.29	0.21	-0.43	-0.58	0.22	0.21	0.22	0.40	-0.48	0.38	0.48	-0.48
**Octanoic acid**	0.08	-0.03	-0.02	-0.19	0.00	-0.53	0.24	0.01	0.23	-0.18	0.05	-0.24	0.10	0.04	-0.16	-0.13	0.01	-0.17	-0.17	-0.18	-0.18	0.12	-0.24	0.08	0.02	-0.22
**Ethyl propanoate**	0.13	-0.10	0.15	-0.20	0.18	0.13	-0.30	-0.19	0.12	-0.37	0.16	-0.15	0.16	0.19	-0.24	-0.14	0.14	0.08	-0.12	-0.12	-0.12	-0.08	-0.06	-0.11	-0.21	0.19
**Ethyl isobutyrate**	-0.26	0.19	-0.30	0.30	-0.34	0.36	-0.05	0.40	-0.28	0.12	-0.01	0.12	-0.01	0.37	-0.50	-0.22	0.01	-0.30	0.26	0.26	0.26	0.21	-0.10	0.18	0.26	-0.46
**Ethyl butyrate**	0.02	-0.06	0.00	0.04	-0.03	0.33	-0.22	0.00	0.07	-0.22	0.15	0.03	0.12	0.31	-0.28	-0.17	0.17	0.02	0.05	0.05	0.05	-0.05	-0.02	0.07	-0.06	0.03
**Ethyl 2-methylbutyrate**	-0.10	0.02	-0.41	0.37	-0.38	0.37	-0.15	0.28	-0.14	0.17	-0.34	0.29	-0.31	0.07	-0.48	-0.03	-0.34	-0.34	0.42	0.42	0.42	0.22	-0.16	0.43	0.44	-0.43
**Ethyl 3-methylbutyrate**	0.10	-0.20	-0.42	0.10	-0.34	0.28	-0.10	0.20	-0.03	-0.04	-0.09	0.17	-0.11	0.43	-0.21	0.02	-0.05	-0.44	0.18	0.17	0.17	0.40	-0.42	0.14	0.26	-0.33
**Ethyl hexanoate**	0.19	-0.28	-0.42	0.25	-0.28	0.28	-0.49	0.21	0.16	-0.13	-0.35	0.48	-0.40	0.20	-0.25	0.17	-0.29	-0.55	0.48	0.47	0.48	0.40	-0.26	-0.04	0.43	-0.23
**Ethyl octanoate**	-0.22	0.18	-0.39	0.36	-0.39	0.10	0.06	0.32	-0.20	0.23	-0.35	0.25	-0.31	-0.12	0.01	0.01	-0.33	-0.30	0.36	0.36	0.36	0.32	0.04	0.37	0.42	-0.39
**Ethyl nonanoate**	0.20	-0.33	-0.78	0.60	-0.78	0.15	-0.27	0.33	0.06	0.18	-0.64	0.49	-0.62	-0.06	-0.56	0.27	-0.64	-0.67	0.60	0.59	0.60	0.42	-0.56	0.18	0.76	-0.63
**Ethyl 2-furoate**	0.26	-0.30	-0.50	0.37	-0.55	-0.19	-0.12	0.12	0.18	-0.04	-0.37	0.28	-0.38	-0.11	-0.55	0.22	-0.39	-0.48	0.28	0.27	0.28	0.22	-0.48	0.05	0.50	-0.48
**Ethyl decanoate**	0.03	-0.10	-0.50	0.40	-0.43	0.19	-0.47	0.23	-0.01	0.03	-0.43	0.27	-0.37	0.25	0.03	0.05	-0.38	-0.43	0.48	0.48	0.48	0.56	-0.17	0.29	0.45	-0.33
**Methyl acetate**	-0.03	0.01	-0.17	0.12	-0.22	0.31	-0.38	0.13	-0.07	-0.27	0.16	-0.06	0.19	0.66	-0.12	-0.27	0.21	-0.15	0.09	0.09	0.09	0.25	-0.14	0.00	-0.03	-0.16
**Propyl acetate**	-0.15	0.06	-0.46	0.36	-0.44	0.12	0.15	0.43	-0.21	0.43	-0.33	0.37	-0.35	-0.11	-0.48	0.08	-0.33	-0.38	0.40	0.40	0.40	0.14	-0.25	0.17	0.54	-0.58
**Isobutyl acetate**	-0.08	0.05	-0.28	0.30	-0.35	0.32	-0.21	0.18	-0.20	0.02	0.03	0.07	0.05	0.28	-0.52	-0.10	0.03	-0.11	0.18	0.18	0.18	-0.01	-0.19	0.06	0.19	-0.35
**Butyl acetate**	0.02	-0.14	-0.67	0.48	-0.60	0.08	0.10	0.37	-0.11	0.42	-0.65	0.61	-0.70	-0.30	-0.43	0.30	-0.64	-0.63	0.58	0.57	0.58	0.41	-0.39	0.48	0.78	-0.62
**Isoamyl acetate**	-0.10	0.04	-0.44	0.23	-0.42	0.10	0.03	0.32	-0.11	0.11	-0.31	0.05	-0.22	0.14	-0.45	-0.15	-0.33	-0.40	0.28	0.27	0.28	0.40	-0.25	0.50	0.38	-0.50
**Hexyl acetate**	0.03	-0.06	-0.28	0.33	-0.31	0.15	-0.44	0.17	0.15	-0.17	-0.12	0.22	-0.12	0.27	-0.18	-0.05	-0.08	-0.23	0.32	0.32	0.32	0.20	-0.04	-0.05	0.26	-0.25
***cis*-3-Hexen-1-yl acetate**	-0.03	-0.02	-0.56	0.47	-0.62	-0.01	-0.21	0.38	0.03	-0.01	-0.33	0.17	-0.25	0.23	-0.61	-0.13	-0.34	-0.49	0.42	0.41	0.42	0.37	-0.32	0.23	0.52	-0.65
***trans*-3-Hexen-1-yl acetate**	0.01	-0.06	-0.41	0.36	-0.48	0.04	-0.17	0.28	0.15	-0.13	-0.13	0.21	-0.14	0.28	-0.51	-0.12	-0.12	-0.36	0.31	0.30	0.30	0.21	-0.23	0.06	0.38	-0.50
**2-Phenethyl acetate**	0.14	-0.14	-0.44	0.33	-0.46	-0.35	0.04	0.11	0.10	0.04	-0.45	0.19	-0.40	-0.24	-0.39	0.18	-0.48	-0.46	0.27	0.27	0.27	0.31	-0.39	0.32	0.51	-0.49
**Methyl decanoate**	-0.05	-0.03	-0.60	0.45	-0.53	0.10	-0.27	0.38	-0.02	0.12	-0.48	0.29	-0.41	0.19	-0.15	0.03	-0.45	-0.57	0.52	0.52	0.52	0.59	-0.21	0.34	0.59	-0.56
**Diethyl succinate**	0.52	-0.45	0.13	-0.27	0.12	-0.58	-0.03	-0.42	0.51	-0.46	0.08	-0.19	0.07	-0.17	-0.19	0.23	0.04	-0.07	-0.33	-0.34	-0.34	-0.16	-0.40	-0.28	-0.13	0.14
**Isoamyl decanoate**	0.24	-0.21	-0.19	0.08	-0.21	-0.31	-0.13	-0.08	0.21	-0.19	-0.14	0.03	-0.11	-0.03	-0.19	0.14	-0.15	-0.27	0.03	0.03	0.03	0.13	-0.30	-0.10	0.17	-0.18
